# The HOG signal pathway contributes to survival strategies of the piezo-tolerant fungus *Aspergillus sydowii* DM1 in hadal sediments

**DOI:** 10.1128/aem.00921-25

**Published:** 2025-08-12

**Authors:** Guangzhao Hu, Maosheng Zhong, Changhao Zhang, Hongfu Lai, Eva Breyer, Jiasong Fang, Xi Yu

**Affiliations:** 1 Shanghai Engineering Research Center of Hadal Science and Technology, College of Oceanography and Ecological Science, Shanghai Ocean University 74595, Shanghai, China; University of Delaware, Lewes, Delaware, USA

**Keywords:** hadal fungi, genome analysis, gene knockout, HOG, HHP

## Abstract

The hadal zone, one of Earth’s most extreme ecosystems, harbors diverse and unique microbial communities adapted to its harsh environmental conditions, including high hydrostatic pressure (HHP) and low temperatures. Within these communities, deep-sea fungi play a critical role in geochemical cycling and marine ecosystem functioning; however, research on their cultivable strains and adaptation mechanism remains scarce. In this study, the piezo-tolerant fungus *Aspergillus sydowii* DM1, isolated from the Mariana Trench sediments (10,898 m), was selected as a representative strain. A comprehensive genome analysis using high-throughput sequencing revealed a genome size of 34.5 Mb, with 12,241 predicted genes. Functional annotations across multiple databases identified a substantial number of pathways associated with environmental adaptations, including extensive carbohydrate, amino acid, sulfur, and nitrogen metabolic pathways. Among them, the HOG (high-osmolarity glycerol) signal pathway, which responds to external stimuli, was indicated to play a crucial role. To study the HOG signal pathways in more detail, we developed a knockout technique for *A. sydowii* and constructed a *hog1* mutant strain (Δ*Ashog1*). The Δ*Ashog1* strain displayed notable differences in colony phenotype, spore production, secondary metabolites, and oxidative stress tolerance compared to the wild type. Furthermore, the *Ashog1* gene was found to regulate reactive oxygen species (ROS) and ATP levels in response to osmotic pressure and HHP, suggesting a role of *hog1* in the fungal adaptation to this extreme environment. Our study serves as an ideal candidate for exploring gene functions in extreme microorganisms and carries significant implications for understanding the adaptive mechanisms of hadal microorganisms.

**IMPORTANCE:**

Research on the genomes and gene functions of hadal zone fungi is crucial for understanding life’s adaptation to extreme environments. However, current studies on constructing genetic operation systems for marine-derived filamentous fungi are scarce, and research on HHP environments in related fields is virtually non-existent. Our study highlights the critical role of the HOG-mediated pathway in regulating stress responses and metabolic processes in extremophiles, a regulatory mechanism that had not been previously investigated under HHP conditions. Notably, the whole-genome annotation of the hadal fungus *Aspergillus sydowii* DM1 advances our understanding of the life processes of hadal fungi. The development of gene knockout technology, combined with insights into stress adaptation and metabolic regulation in *A. sydowii* strain DM1, provides a strong foundation for future research and biotechnological applications.

## INTRODUCTION

The hadal ecosystem represents one of the most extreme environments on Earth, characterized by unique biodiversity including a vast array of largely unexplored microorganisms (1). The hadal zone (> 6,000  m depth of the ocean) is characterized by high pressure, low temperature, complete darkness, and limited nutrient availability (2, 3). Although under these harsh conditions, hadal microbes still have strong biological activity and play an important ecological role, such as participating in element cycling and the decomposition of organic matter (3–5). Recent studies on hadal microorganisms have uncovered specialized metabolic pathways and genomic features tailored to these harsh conditions. In extreme cases, microbes adapt their genomes to respond to their environment, such as the shrinking genome of bacteria found in the gut of deep-sea amphipods (6). Fungi isolated from the Challenger Deep exhibit complete dissimilatory and assimilatory nitrate reduction pathways, along with genes associated with partial denitrification (7). These adaptations illustrate the capacity of life to thrive under intense environmental stress.

As a significant characteristic of the hadal environment, HHP exerts a profound influence on the biological processes of microorganisms, such as transcription, translation, membrane composition, and proteins (8–10). In the field of food sterilization, the role of ultra-high pressure (exceeding 300 MPa) on *Saccharomyces cerevisiae,* a typical model yeast, has been investigated, showing HHP disrupted the cell membrane and cell wall structures and inactivated proteins, DNA, and other substances (11–13). At *in situ* conditions, HHP has the potential to activate the synthesis of certain amino (9, 14) and can lead to redox imbalance and insufficient energy supply in cells (15). Furthermore, previous studies demonstrated that HHP (> 20 MPa) could affect the growth and synthesis of secondary metabolites of hadal fungi and trigger high expression of related genes (16, 17). The impact of HHP on the transcriptome of hadal fungi had been analyzed, revealing that fungi respond to high pressure by enhancing the cell wall structure, lipid and fatty acid biosynthesis, while reducing the cell cycle and energy metabolism (18, 19). However, currently, there is still a lack of targeted research specifically focusing on the functional genes involved in HHP adaptation of hadal fungi.

The extreme conditions of the hadal environment pose significant challenges to the organisms therein (3). The signaling pathways of eukaryotic cells play a crucial role in responding to environmental changes, among which the MAPKs (mitogen-activated protein kinases) signal pathway is particularly important (20). Hog1 is a core member of the MAPK family, highly conserved across fungi, and plays an important role in regulating multiple cellular physiological processes (21). Initially identified in Saccharomyces cerevisiae, *hog1* is activated by osmotic stress, enabling cells to maintain internal pressure by activating the corresponding genes (GPD2, RHR2) (22). Previous studies have demonstrated that *hog1* also inhibits hyphal growth (23), cell wall synthesis (24), and regulates the cell cycle and protein turnover (25, 26). Additionally, signals such as oxidative stress and DNA-damaging agents can activate the pathway (27, 28). In the high osmotic pressure’s environment of the ocean, the *hog1* gene plays a crucial role in maintaining osmotic pressure (29, 30). The results of these studies suggest that, given the ability of *hog1* to respond to a wide range of environmental stress stimuli, it may also play a significant role in the extreme hadal environment. However, functional genetic studies employing knockout techniques remain limited in marine fungal research, particularly for hadal fungi. The role of *hog1* in HHP adaptation in hadal fungi represents a significant knowledge gap.

*Aspergillus* spp. are widely distributed in marine environments, including deep-sea sediments and abyssal zones, as evidenced by representative isolations from diverse oceanic habitats (7, 31, 32). In our previous study, transcriptome analysis revealed that the expression pattern of the HOG system may play a critical role in modulating the high-pressure response of the hadal fungus *Aspergillus sydowii*, particularly in regulating cellular energy metabolism and the cell division cycle (18). An efficient genetic manipulation system is essential for establishing a foundation for subsequent gene function studies. Here, we demonstrate the genomic characteristics of *A. sydowii* DM1. Specifically, we successfully performed *hog1* knockout experiments, demonstrating that the *hog1* is involved in energy metabolism and oxidative stress responses to osmotic pressure and HHP in hadal fungi. The construction of this model strain DM1 and the detailed investigation of its gene functions will advance our understanding of how hadal microorganisms survive in extreme environments, potentially revealing novel biochemical pathways and stress resistance mechanisms.

## RESULTS

### The genomic annotation of DM1 contains features adapted to the hadal environment

In our previous study, *A. sydowii* DM1, isolated from sediments at a depth of 10,898 m in the Mariana Trench, was identified as exhibiting notable piezo-tolerance (18). To gain a deeper understanding of its adaptative mechanism, here, the genome was sequenced. The complete genome of strain DM1 comprised 21 contigs, with an N50 of 2.6 Mb, a GC content of 50.57%, and a total size of 34.5 Mb. The maximum contig length was 5,244,598 base pairs. Gene prediction and completeness evaluation identified a total of 12,241 genes, Fig. S1 and S2). The overall genomic characteristics were comparable to those of the five strains of *A. sydowii* currently available in the NCBI database (Table S1 to S3). This number was lower than that of the shallow-sea strain CBS 593.65, which has 13,579 genes, and the deep-sea strain BOBA1, which has 14,103 genes from a depth of 3,000 m.

**Fig 1:**
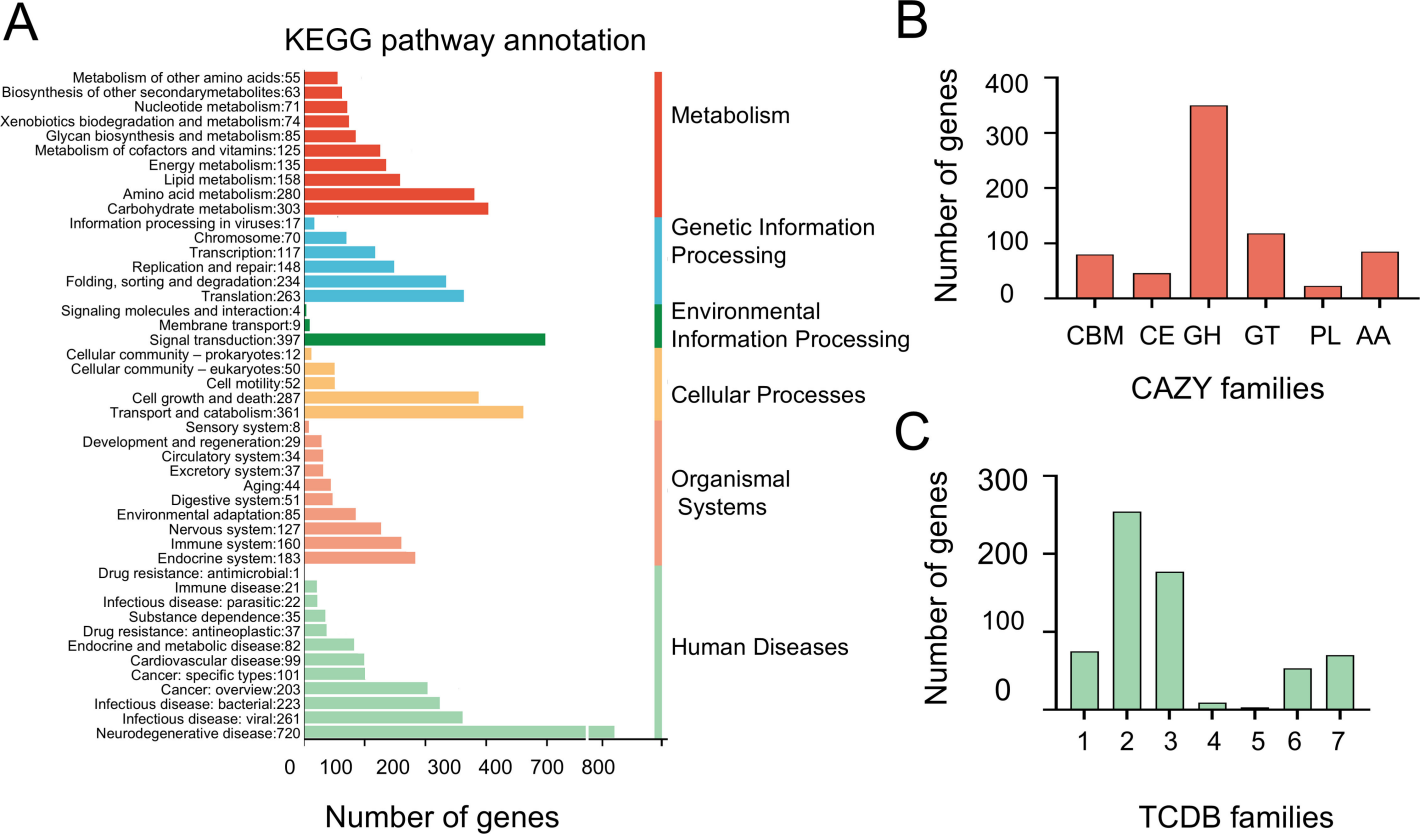
DM1 genome annotation via multiple databases. The specific quantity is indicated in parentheses. (A) KEGG pathway of *A. sydowii* DM1. (B) CAZy annotations of DM1. CBM, carbohydrate-binding modules (80); CE, carbohydrate esterases (46); GH, glycoside hydrolases (350); GT, glycosyl transferases (118); PL, polysaccharide lyases (23); AA, auxiliary activities (85). C TCDB function classification of DM1: 1, Channels/Pores (75); 2, Electrochemical Potential-Driven Transporters (254); 3, Primary Active Transporters (177); 4, Group Translocators (9); 5, Transmembrane Electron Carriers (3); 6, Accessory Factors Involved in Transport (53); 7, Incompletely Characterized Transport Systems (70).

The *A. sydowii* DM1’s genome was annotated using multiple databases. The genome annotation revealed 7,558 genes (61.7%) in the KEGG (Kyoto Encyclopedia of Genes and Genomes) (33) database (Fig. 1A). For BOBA1, 7,960 genes (56.4%) were annotated, while for CBS 593.65, 7,867 genes (57.9%) were annotated (Table S4). Among these pathway annotation, those related to carbohydrate metabolism and amino acid metabolism indicated that strain *A. sydowii* DM1 could utilize various carbon sources and had the potential for amino acid synthesis and metabolism. Regarding genetic processes, the translation pathway predicted the highest number of genes (263), while 148 genes were identified in the replication/repair pathway and 234 in the folding/sorting/degradation pathway. The complete results, in comparison with the KEGG analysis of the other two strains, were provided in the supplementary material (Table S4). Our analysis revealed that DM1 exhibited a higher relative abundance in several metabolic pathways, including “Replication and repair,” “Chromosome,” “Transport and catabolism,” and “Cell growth and death” compared to the other two strains of *Aspergillus*.

According to the CAZy database (Fig. 1B), the DM1 genome contained 80 CBMs, 46 CEs, 350 glycosidases or GHs, 118 GTs, 23 PLs, and 85 AAs, which were annotated for their roles in the degradation of chitin, lignin, and microplastics. Furthermore, analysis using the TCDB database revealed 89 distinct transporter gene types in the DM1 genome (Fig. 1C) responsible for transporting carbohydrates, various metal ions, ammonia, sulfides, and other substances. In terms of carbon metabolism, strain *A. sydowii* DM1 exhibited complete pathways for glycolysis/gluconeogenesis, the citric acid cycle, and the pentose phosphate pathway. The annotation results related to KOG and the Gene Ontology (GO) database were shown in Fig. S3 and S4.

Clustering analysis identified 38 unique genes in strain *A. sydowii* DM1 (Table S5), which were absent in the other two *Aspergillus* strains. Annotation analysis using eggNOG (Evolutionary Genealogy of Genes: Non-supervised Orthologous Groups) revealed functional annotations for 24 of these genes. Among these, four genes were associated with the function “Pkinase” and eight genes were linked to the “HCO_3_^-^ transporter” function, as annotated in the Pfam annotation database. These genes were indicated to play a role in the adaptation mechanisms to the hadal environment. Notably, a significant portion of the unique genes remains unannotated, and further functional studies of these genes may provide valuable insights.

### Constructing *A. sydowii* DM1 metabolic network

**Fig 2:**
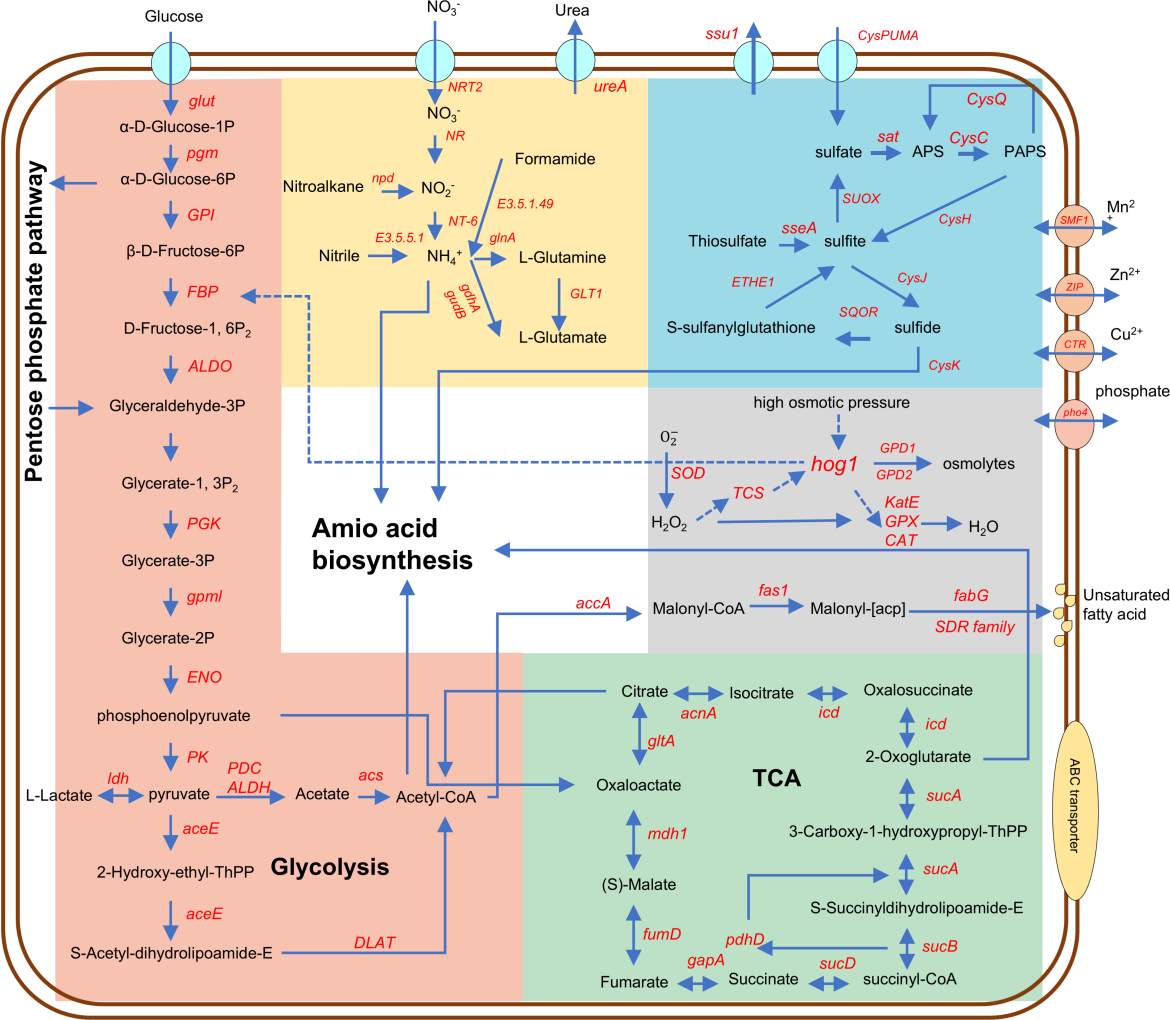
Predicted metabolic potential of hadal-derived fungus *A. sydowii* DM1 from genomic data. The arrows within the figure show the predicted pathway, and the dashed arrows represent the regulation of gene expression.

Using functional annotations from KEGG, TCDB, and other databases, we predicted gene functions in *A. sydowii* DM1 and reconstructed its metabolic pathway network. These genes were functionally associated with key carbohydrate metabolism pathways, including glycolysis/gluconeogenesis, oxidative phosphorylation, the pentose phosphate pathway (soft orange, Fig. 2), pyruvate metabolism, and tricarboxylic acid (TCA) cycle (green, Fig. 2). In sulfur metabolism, genomic analysis revealed that *A. sydowii* DM1 contains a complete assimilatory sulfate reduction pathway, some core components of the sulfur oxidation (SOX) system, and a dissimilatory sulfate reduction pathway (blue, Fig. 2). The presence of these pathways suggests that *A. sydowii* DM1 is capable of utilizing sulfate reduction to generate energy in low-oxygen or anaerobic conditions. Additionally, the SOX system indicates the potential for the oxidation of thiosulfates and sulfides.

Regarding nitrogen metabolism, the genome of *A. sydowii* DM1 contained a complete nitrate reduction pathway (yellow, Fig. 2), including the genes *NRT*, *NR*, and *NT-6*, as well as three key genes for ammonia assimilation: *glnA*, *GLT1*, and *gdhA*. This suggests that *A. sydowii* DM1 may play a role in the marine nitrogen cycle, with nitrate serving as the terminal electron acceptor in anaerobic respiration (denitrification). Some *Fusarium* species in hadal oxygen-limiting environments, as well as salt-tolerant *Penicillium* and *Aspergillus* species in other low-oxygen habitats like anaerobic marine sediments, also possess the ability to carry out the denitrification process (34). These findings further imply that *A. sydowii* DM1 might possess adaptive mechanisms enabling its survival and proliferation in hadal environments characterized by low oxygen levels.

In addition, studies have shown that HHP affects the permeability of cell membranes (35, 36). The presence of a complete fatty acid β-oxidation pathway in DM1 suggested that this strain can maintain cell membrane fluidity by synthesizing unsaturated fatty acids under high hydrostatic pressure, thereby preserving the stability of internal cellular structures. In terms of HHP tolerance, key genes associated with oxidative stress—such as those encoding superoxide dismutase activity and ROS scavengers (*sod*, *CAT, GPX*), were identified in the genome of *A. sydowii* DM1 (gray, Fig. 2), which were functionally regulated by HOG pathway. Additionally, genes involved in tryptophan biosynthesis were also detected, which contributes to enhances the growth of brewing yeast under HHP, up to 25 MPa (37).

### Construction and characterization of mutant strain

In our previous research on the transcriptomes of the hadal strain *A. sydowii* DM1 under HHP conditions, *hog1* was involved in the regulation of the series of responses exhibited by fungal cells in resisting HHP stimulation. Additionally, it was observed that the expression pattern of the HOG pathway under HHP conditions exhibited similarities to that observed under hyperosmotic conditions (2 M NaCl) (18). To further investigate the function of *hog1*, an efficient genetic manipulation system was established based on homologous recombination.

**Fig 3:**
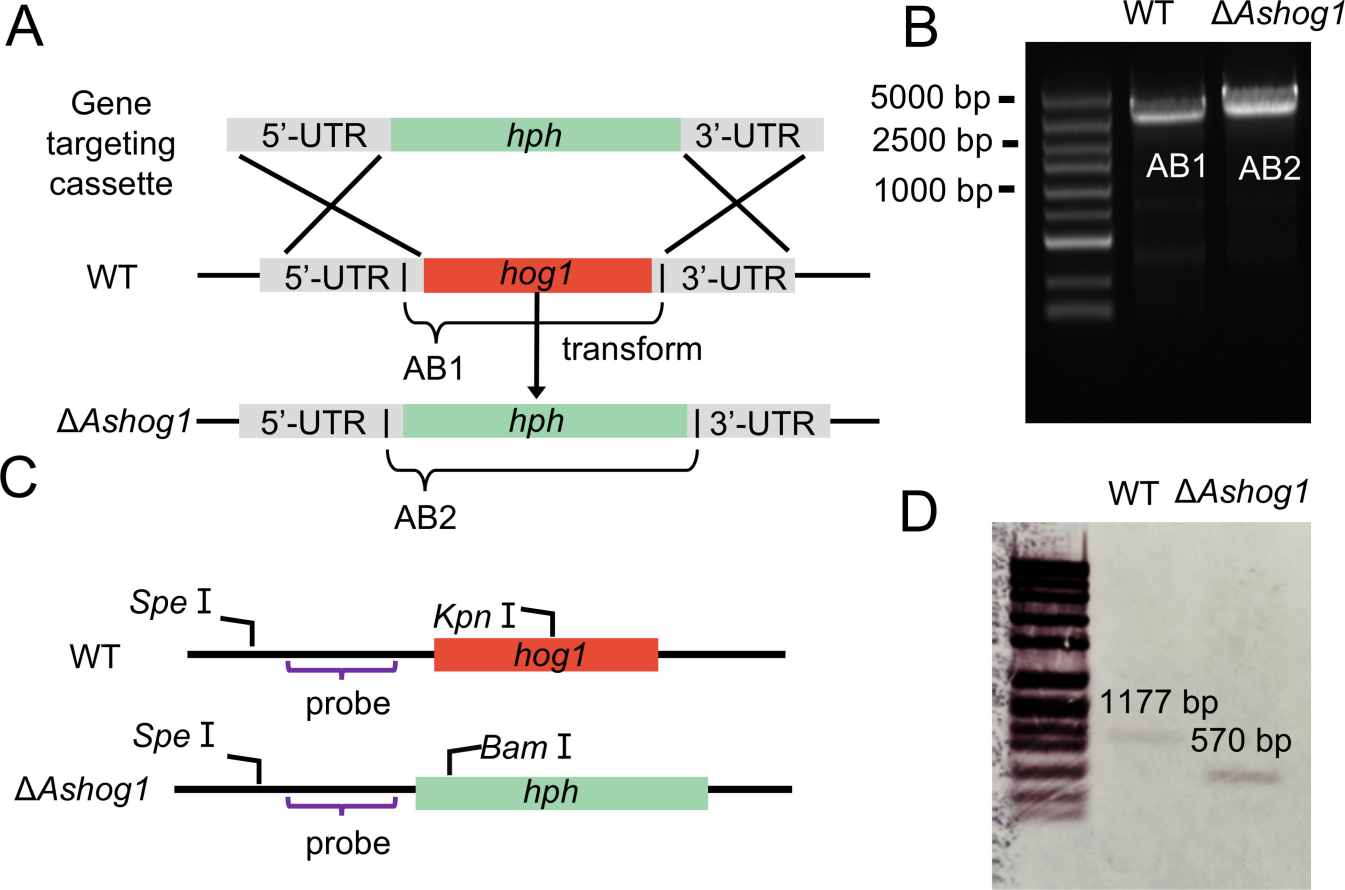
Construction and validation of *hog1* deleted strain by homologous recombination. (A) Deletion of the *hog1* gene using homologous recombination. In the Δ*Ashog1* strain, the *hog1* (1.6 kb) was replaced by the 1.8 kb hygromycin resistance gene *hph*. (B) The mutant strains were validated using PCR. AB1 and AB2 represented the full length from 5′ to 3′, which were 3.2 kb and 3.4 kb, respectively. (C) Southern Blotting analysis for confirming the Δ*Ashog* mutant. (D) Genomic DNA of WT and the Δ*Ashog1* mutant was digested using the *Kpn I* and *Bam I* restriction enzyme resulting in a 0.5 kb fragment after hybridization with the *hph*-probe.

Through the analysis of genomic data obtained from NCBI and previous research, we identified the homologous gene *A3061* of *hog1* in DM1. This gene was 1,653 bp in length and contained 5 introns, with the longest intron measuring 512 bp. The coding sequence of *A3061* spanned 893 bp, and BLAST analysis against the NCBI database revealed a 99.55% similarity to the *hog1* gene of *Aspergillus versicolor*. Thus, we designated this gene as *Ashog1*. To investigate the function of *Ashog1* in *A. sydowii*, we generated *Ashog1* deletion mutants through gene replacement, using the hygromycin B phosphotransferase gene (*hph*) as a selection marker. PCR analysis confirmed the absence of the *Ashog1* fragment in Δ*Ashog1* strains (Fig. S5), and the mutant PCR amplicon length was 3,404 bp which was longer than the 3,204 bp amplicon of the WT strain (Fig. 3B; primer details are provided in Table S6). Additionally, Southern Blotting was performed to validate the successful deletion of *Ashog1* (Fig. 3C and D; Table S7). The WT strain showed a hybridizing band of 1,177 bp, whereas the knockout strain displayed a shorter fragment of 570 bp. This finding further confirms the complete loss of the *Ashog1* gene in the Δ*Ashog1* strain, providing solid evidence for successful gene knockout. The complemented strain was successfully constructed using a similar method (details were provided in the METHODS AND MATERIALS section), and the presence of full-length *hog1* fragments was verified by PCR (Fig. S6).

### Δ*Ashog1* and WT showed differences in colony morphology and secondary metabolites

**Fig 4:**
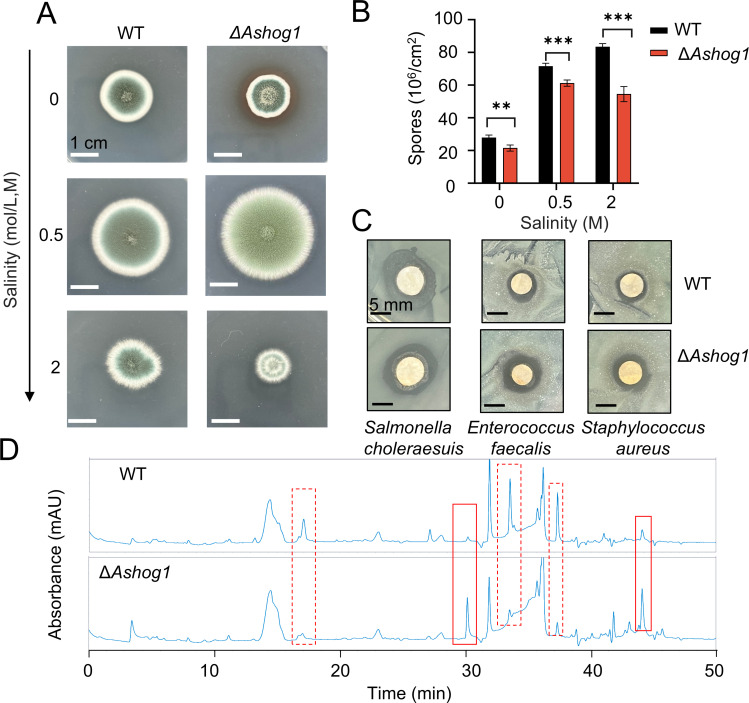
Δ*Ashog1* and WT showed differences in colony morphology and secondary metabolites. (A) Morphology of WT and knockout strains after growing on plates with different salinities for 3 days. Scale bar represents 1 cm. (B) Comparison of spore numbers of WT and knockout strains grown on PDA (potato dextrose agar) with different salinities for 5 days. Asterisks represent statistically significant differences determined by one-way ANOVA (n.s. *P* > 0.05, *P < 0.1, **P < 0.01, ***P < 0.001, and ****P < 0.0001). (C) Zones of inhibition of the metabolites produced by WT and knockout strains. (D) Comparison of HPLC spectra between WT and knockout strains. The image above is from the WT, and the image below is from the knockout mutant. The solid-line boxes and dashed-line boxes represent the peaks that increased and decreased in the Δ*Ashog1* compared to the WT strains.

We additionally investigated the effect of *Ashog1* on phenotype, spore production, and secondary metabolite synthesis. Morphologically, the Δ*Ashog1* strain exhibited significant differences compared to the WT strain (Fig. 4A). To determine the roles of *hog1* under osmotic stress, the phenotype of WT and mutant was detected. In response to varying salinity levels, both the knockout strain and the WT demonstrated disparate growth patterns. Among the conditions tested, Δ*Ashog1* showed the fastest growth at a salinity of 0.5 M (Fig. 4A), which represents the optimal salinity in ocean condition. By contrast, the growth rates of the mutant were significantly reduced under hypotonic or hypertonic conditions due to the osmoregulation and osmotic stress response. While the complemented strain exhibited a restored phenotype comparable to that of the wild type when cultured in 0.5 M NaCl (Fig. 7A). The Δ*Ashog1* strain exhibited a reduction in spore number compared to the WT strain. Specifically, the spore count decreased by 22.85% at a concentration of 0 M NaCl, by 14.4% at a concentration of 0.5 M NaCl, and by 34.7% at a concentration of 2 M NaCl (Fig. 4B). The color of the knockout strain plate at the back intensified in comparison to the WT strain under 0 M conditions. Differences in coloration behind the culture plates suggested potential variations in secondary metabolite production, indicating an increase in secondary metabolites. To further investigate the biological function of *hog1*, we extracted secondary metabolites from both strains and analyzed them using HPLC (Fig. 4D). Distinct differences were observed in the chromatograms, as indicated by the red boxes. The knockout strain showed a significant reduction in three peaks (solid box) and an increase in two peaks (dashed box), indicating a regulatory role of *Ashog1* in secondary metabolites production. Additionally, we conducted inhibition zone assays using the crude product (100  mg/mL) and found differences in its inhibitory activity against three pathogenic bacteria (*Salmonella choleraesuis, Enterococcus faecalis, Staphylococcus aureus*) (Fig. 4C), demonstrating that the observed changes in the antibacterial products.

### *Ashog1* positively regulates oxidative stress tolerance

**Fig 5:**
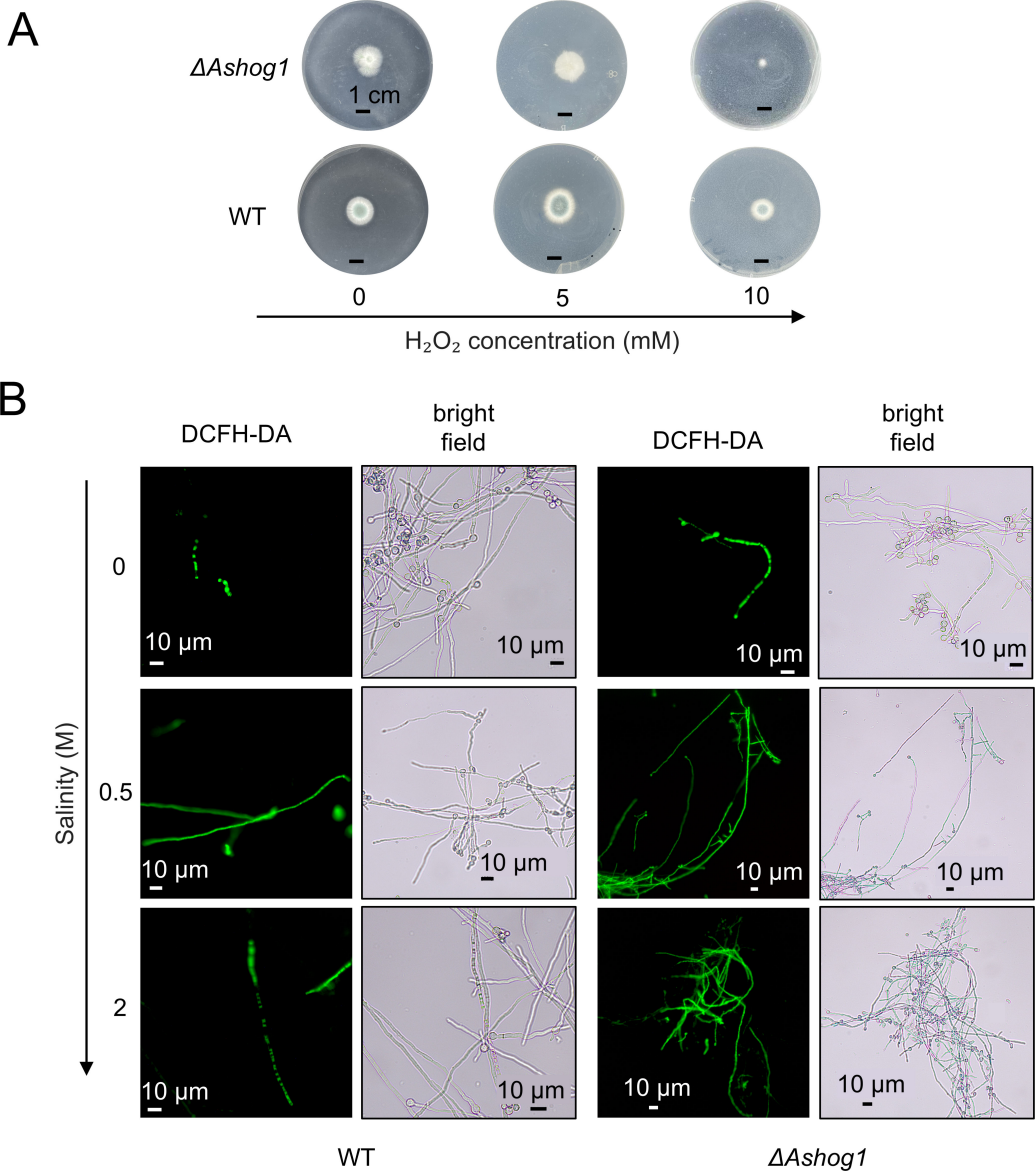
The response of two strains to oxidative stress. (A) The growth of WT and knockout strains on PDA containing different concentrations of hydrogen peroxide after 3 days. (B) ROS staining of hyphae cultured in PDB at different salinities. The scale bar represents 10 µm.

Studies have shown that *hog1* mediates the oxidative stress pathway of *Candida albicans* (38). The conidia of WT and Δ *Ashog1* (1 ×  10^6^ spores/mL) were cultured on PDA with different concentrations of H_2_O_2_ (5 mM, 10 mM, 20 mM, 30 mM, 50 mM) to verify whether *Ashog1* of hadal fungus was involved in oxidative stress. We observed that at the concentration of 10 mM, the diameter of Δ*Ashog1* colony decreased and the edge was irregular (Fig. 5A), indicating that the tolerance of Δ*Ashog1* to oxidative stress was significantly reduced. Especially on the third day, the colonies hardly grew. However, there was no significant difference in colony growth at 5 mM. The colonies could not be detected when the concentration of hydrogen peroxide was greater than or equal to 20 mM.

ROS play a pivotal role in fungal physiology, functioning as signaling molecules that regulate various cellular processes, including growth, differentiation, and stress responses (39–41). Environmental stressors, such as high salinity, can exacerbate ROS production, leading to oxidative stress within fungal cells (42, 43). In this study, ROS levels were measured under three salt concentrations: 0, 0.5, and 2 M, respectively. The green fluorescence of the Δ*Ashog1* strain hyphae was significantly higher than that of the WT under all three salt concentrations (Fig. 5B). This indicates that the fungus’s ability to process ROS decreased after losing the regulation of the *Ashog1* gene, resulting in an increased ROS concentration. The ROS level of the Δ*Ashog1* strain’s hyphae was always higher than that of the WT strain. Our results indicated that *Ashog1* was activated in response to elevated H₂O₂ levels and high salinity, triggering signaling cascades which effectively reduced intracellular ROS levels. This reduction might help the cells to adapt to these adverse conditions. The ROS level of the complement strain under 0.5 M NaCl was restored to the WT level (Fig. S7), confirming the role of *Ashog1* in ROS regulation.

### *Ashog1* exhibits regulatory functions on cell viability and ROS at osmotic pressures

**Fig 6:**
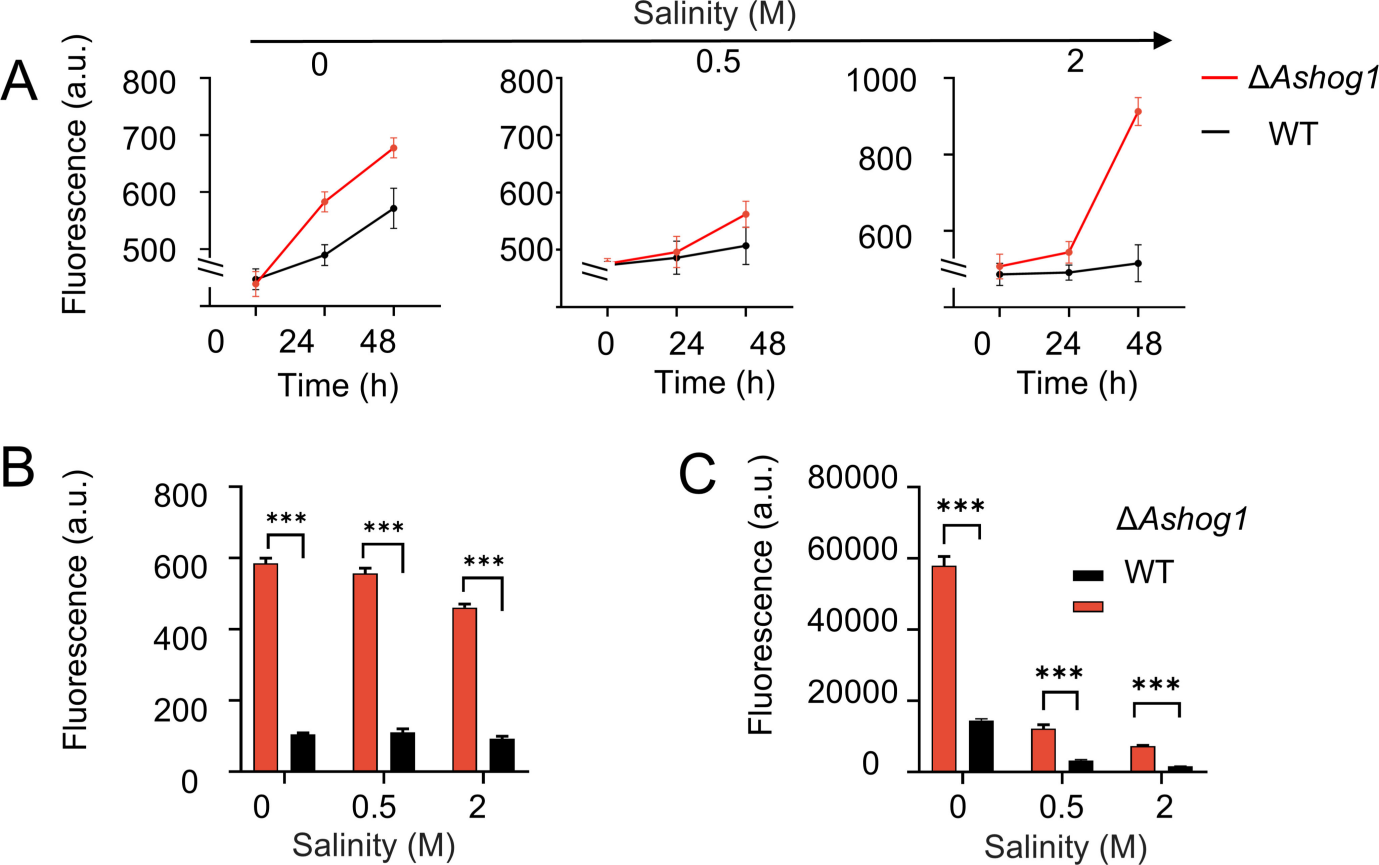
Comparison of spore activity and ROS levels between the two strains at different salinities. The spore suspension was obtained from fungi which grew in PDAs with different salinities. (A) The changes of the fluorescence value of resazurin in spore suspension under three salt concentrations were measured every 24 h (25°C, dark). (B) ROS measurement of spore suspension under different salt concentrations. (C) ATP measurement of spore suspension at different salinities.

Resazurin, a redox indicator commonly used to assess cellular respiration and viability, is reduced to resorufin in the presence of active mitochondrial respiration, reflecting increased metabolic activity (44). ATP levels serve as a critical measure of cellular energy status, with higher ATP concentrations indicating elevated metabolic processes and energy availability (45). In our study, we observed that the Δ*Ashog1* strain exhibited higher resazurin and elevated ATP levels compared to the WT strain. Under 0, 0.5, and 2 M of NaCl, the metabolic activity of Δ*Ashog1* was 1.18, 1.10, and 1.77 times higher than that of the WT (Fig. 6A). This suggests that the absence of *Ashog1* leads to increased cellular metabolic activity, with the greatest discrepancy observed under the 2 M NaCl condition. The measurement results for ROS were not significantly different under varying salinity conditions. However, a notable discrepancy was observed between the Δ*Ashog1* and WT strains, with the former exhibiting ROS levels that were 5.61, 5.05, and 5.01 times higher than the latter at the three salt concentrations (Fig. 6B). The results of the ATP measurement indicate a decrease in the measured values with an increase in salinity. Furthermore, the ATP and ROS level of Δ*Ashog1* were found to be higher than those of the WT. The measured ATP levels of Δ*Ashog1* at the three salt concentrations were 4.00, 3.82, and 4.53 times that of WT, respectively (Fig. 6C). The elevated resazurin result indicates heightened respiration rates, while the increased ATP levels reflect greater energy production. These findings imply that *Ashog1*, which is involved in regulating stress responses and cell cycle progression, may influence cellular metabolism. Without *Ashog1*, cells could bypass certain stress response checkpoints, leading to enhanced metabolic activity and energy production, which may ultimately result in more rapid mycelial growth. This unexpected increase in cellular activity in the Δ*Ashog1* strain highlights the complex role of *Ashog1* in balancing stress adaptation and metabolic regulation.

### *Ashog1* regulated the intracellular ROS and ATP levels under varying HHP conditions

**Fig 7:**
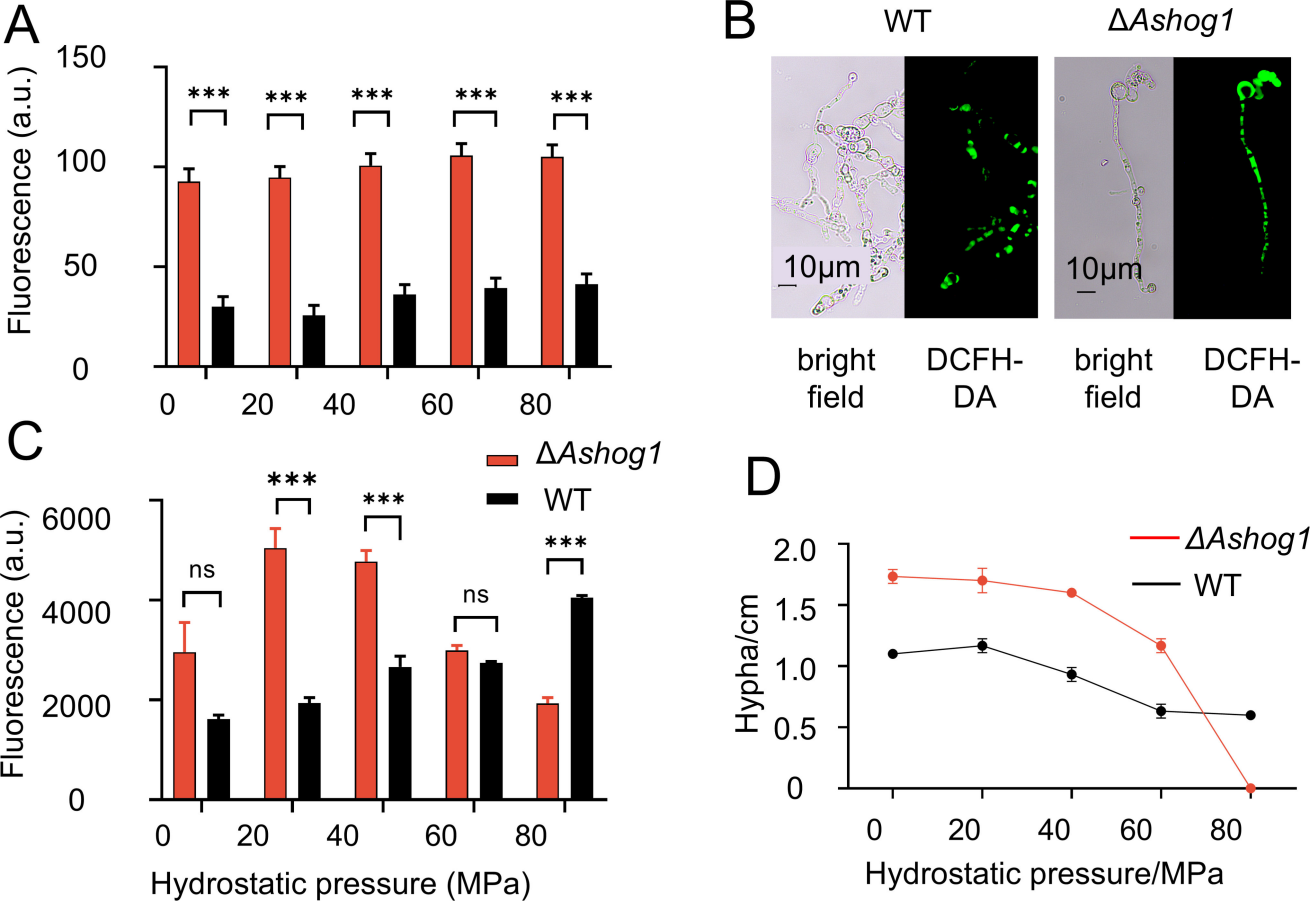
ROS & ATP contents and mycelium growth of two strains under different HHP treatments. (A) ROS variation at 2 days under different pressures. (B) ROS in hyphae of two strains at 20 MPa after 10 h. (C) ATP variation at 2 days under different pressures. (D) Colony diameter after 14-day HHP treatment and 2-day incubation on PDA plates (*n* = 3).

To further investigate the impact of the *Ashog1* gene on fungi living at hadal high-pressure environments, we conducted a preliminary experiment to assess the effects of HHP on WT and Δ*Ashog1* strains. Based on our previous findings, we established four pressure conditions: 20, 40, 60, and 80 MPa, which roughly simulate the pressure conditions from the ocean surface to depths of 8,000 m. After 2 days of pressure treatment, we measured the levels of ROS and ATP in spore suspensions. The ROS levels in the Δ*Ashog1* strains were significantly higher than those in the WT strains across all experimental groups (Fig. 7A), with no obvious changes observed with increasing pressure. The ROS values for the Δ*Ashog1* strain were 93, 97, 101, 106, and 105, while for the WT strain, they were 30, 26, 36, 39, and 41, with a *t*-test showing a significant difference between groups (*P* < 0.0001). Microscopic observations showed that only some parts of the WT mycelia displayed green fluorescence, while the Δ*Ashog1* strain was completely green, indicating higher ROS accumulation (Fig. 7B). In the ATP measurement experiment, the WT and Δ*Ashog1* strains showed divergent responses to increasing pressure (Fig. 7C). The ATP levels in the WT strain demonstrated a gradual increase in response to pressure, indicating that high hydrostatic pressure stimulated cellular energy metabolism. In contrast, the ATP levels in the knockout strain initially increased and then decreased, with the highest levels observed between 20 and 40 MPa. This phenomenon may be attributed to the loss of regulation of the *Ashog1*, which results in accelerated metabolism and increased energy consumption within the spores. Under the HHP treatment condition, the levels of ATP in Δ*Ashog1* decreased rapidly, thereby may leading to difficulties in spore germination. We also observed that the growth rate of the mycelia of Δ*Ashog1* strains was higher than that of WT prior to 80 MPa (Fig. 7D), which might be associated with higher intracellular ATP levels. With the intensification of HHP, the growth rate of Δ*Ashog1* strains decreased to a greater extent than that of WT, suggesting that they were more significantly affected by stress (Fig. 7D). The spores of Δ*Ashog1* within the 80 MPa group failed to germinate on PDA after 2 days. We also performed determination of spore germination in PDB and observed almost no spore germination in the Δ*Ashog1* strains under the condition of 80 MPa after 24 h. This was comparable to the growth observed on the mycelium agar culture (Fig. S8).

## DISCUSSION

As research into hadal fungi has progressed, they have become of increasing interest due to the novelty of their secondary metabolites and the significance of their ecological roles in geochemical cycles (46). The extreme environment exerts a profound influence on the metabolic capacity and functional genes of hadal microorganisms (17, 19, 36). However, current research on hadal microbial genes has mainly focused on metagenomic analysis. There is a gap of knowledge for molecular research on culturable isolates located below 6,000 m. Furthermore, the lack of a targeted gene knockout method has resulted in a scarcity of research into the role of key genes in adapting to HHP. Our study presents a comprehensive genomic analysis of *A. sydowii* DM1, a culturable filamentous fungus isolated from the hadal zone, revealing unique genomic signatures and functional adaptations to extreme deep-sea environments. Notably, comparative KEGG pathway annotation revealed a significant enrichment in genes associated with “Replication and repair,” “Transport and catabolism,” and “Cell growth and death,” suggesting these metabolic and regulatory features may represent key genomic adaptations of hadal fungi to the extreme conditions. While genetic manipulation systems are well-established for terrestrial fungi, their application in marine fungi—particularly deep-sea species—remains technically challenging, such as resistance to conventional antibiotics and inefficient transformation protocols. In this study, we successfully developed a targeted gene knockout system for a hadal-zone fungal strain. Although the current methodology still has limitations and requires further optimization, such as the need for prior disruption of Δ*ku70* to enhance homologous recombination or the potential optimization using CRISPR/Cas9-based systems (47, 48), it represents a substantial advancement in establishing genetic operating systems for marine fungi, also opens new avenues for investigating their ecological roles and adaptive mechanisms in extreme environments.

*Aspergillus* spp. have a wide global distribution and are one of the most dominant fungi in the ocean (49). Among these, *A. sydowii* has been found in environments with high salinity and pressure indicating its adaptability to diverse environmental conditions (32). Ganesh Kumar and others studied the genome of *A. sydowii* collected at a depth of 3,000 m and found genes related to deep-sea survival such as DNA repair and oxidoreductases which enables them to protect themselves from multiple stressors (50). Here, our genomic analysis reveals that the hadal fungus *A. sydowii* DM1 maintains expanded metabolic gene clusters to indicate the strain’s adaptability. The complete assimilatory sulfate reduction pathway in DM1 implies an adaptive strategy to circumvent energy limitations in oxygen-depleted hadal environments, analogous to sulfate-reducing bacteria (7). These genomic features in the hadal strain DM1 may contribute to the environmental adaptation of hadal fungi to extreme conditions. However, current investigations into microbial high-pressure adaptation mechanisms remain predominantly bioinformatics-based, with limited experimental evidence to substantiate the findings (19, 36, 51). Hence, the functional validation through gene knockout experiments in future is critical to establish causal relationships.

The HOG-MAPK signaling pathway, mediated by the core protein kinase HOG, is a well-known signaling cascade that plays a role in osmotic pressure regulation, mycelial morphology, and oxidative stress of fungi (28, 38, 52–55). In deep-sea, previous studies have demonstrated a close association between microbial high-pressure adaptation mechanisms and oxidative stress, as well as ROS production (36, 56). That raises the question if HOG pathway mediates the activity of HHP tolerance in deep-sea fungi. Our results suggest that *Ashog1* was essential for regulating stress responses, developmental processes, and metabolite production in *A. sydowii*, extending the conserved HOG functions to high-pressure conditions previously unexamined in fungi (28). These findings expand our understanding of HOG pathway functions beyond terrestrial systems and highlight its potential ecological significance in marine fungi. Furthermore, our study revealed the influence of the HOG pathway on secondary metabolites. Although we did not identify specific secondary metabolites, the observed differential expression patterns suggest that the HOG pathway may modulate metabolite production as part of fungal adaptation to deep-sea environments. Elevated levels of ROS in the Δ*Ashog1* strain indicated that ROS regulation was impaired, indicating that the *Ashog1* gene plays a crucial role in the management of oxidative stress, which is exacerbated under HHP. Excessive ROS are harmful to cells (39, 41, 43). The elevation of ROS levels might be an important reason for the damage that HHP inflicts on cells. However, the regulation of ROS levels within cells involves complex mechanisms and regulatory networks. In this experiment, the elevation of ROS levels is likely attributable to the synergistic effects of the HHP and HOG pathway, also may be due to other signaling pathways. Another interesting point is that we found *hog1* had a significant impact on ATP levels. ATP levels are important for maintaining cellular physiological functions, but some studies have shown that high ATP levels have the potential to harm cell growth and metabolism, also disrupt protein synthesis and tend to guide cells towards apoptosis (45, 57, 58). To our knowledge, few studies have investigated the effect of *hog1* on ATP levels. Cells with high energy levels were more sensitive to HHP in the absence of *Ashog1* regulation, indicating *Ashog1* sustains cellular activity by regulating ROS levels and metabolic viability. Our study elucidates a previously unrecognized molecular mechanism underlying HHP tolerance in deep-sea fungi, providing new insights into microbial adaptation to extreme environments.

The HOG pathway is a highly conserved gene that is ubiquitously present and extensively studied in microorganisms (59, 60). While the *hog1* is well recognized for its pivotal role in environmental stress adaptation, its function in high-pressure adaptation by marine filamentous fungi remains poorly understood. Our previous data demonstrated that the fungi from different origins universally possess high pressure tolerance, while hadal-derived strains, indeed, exhibited superior tolerance ability, along with differential expression of key oxidative stress-related genes, including genes in HOG pathway, which would be involved in the HHP adaptation mechanism (18). These findings suggest differential regulatory mechanisms of the HOG pathway in *Aspergillus* strains from distinct habitats under HHP conditions, highlighting the need for further investigation into habitat-specific fungal adaptations to high-pressure environments. Combined with the oxidative stress and ATP assays in this study, the role of *hog1* in response to HHP environment stress was validated. That means HOG pathway contributes to the HHP tolerance when fungi face to this extreme environment, while the enhanced tolerance of hadal-derived filamentous fungi likely relies more heavily on the transcriptional regulation of the key genes. Although, more knock-out validation should be conducted in further. Our findings significantly advance this underexplored field, providing critical insights into this mechanistic gap.

## MATERIALS AND METHODS

### Sample acquisition, isolation, and cultivation conditions

Deep-sea fungi were isolated from hadal sediment samples collected at a depth of 10,898 m in the Mariana Trench (142.2148 °E, 11.3403 °N) in October 2021. Details on isolation, purification, and species identification of fungi are published in our previous study (18). Fungal strains were maintained in glycerol stocks at −80°C and re-cultured on PDA (potato dextrose agar) at 28°C for 7 days. Unless otherwise noted, PDA was used as culture medium in this study (media concentrations are shown in Table S8).

### Genome analysis and genome acquisition of two additional *A. sydowii* strains

Annotation was conducted using the NR (61), Gene Ontology (62), and KOG (http://www.ncbi.nlm.nih.gov/COG/) databases. Two genomes of *A. sydowii* strains isolated from the ocean were retrieved from NCBI (https://www.ncbi.nlm.nih.gov/datasets/genome/), specifically Boba1 (GCA_009828905.1, isolated at 3,000 m depth in Bay of Bengal) and CBS 593.65 (GCA_001890705.1, isolated from shallow waters). To ensure credible results, genomic data were acquired and re-annotated. Afterward, cluster analysis was performed and gene structure was predicted using the SPAdes. The quality of the predicted genes was assessed with BUSCO. The homologous proteins in the three genomes were identified using default parameters of OrthoFinder (https://github.com/davidemms/OrthoFinder). Annotation was carried out with eggNOG.

### Genome sequencing and assembly

DNA was extracted with the GP1 method (cetyltrimethylammonium bromide, CTAB) (63). The integrity and purity of the extracted DNA were assessed by 1% agarose gel electrophoresis. DNA concentration was quantified using a Qubit 2.0 Fluorometer (Thermo Scientific). A sequencing library was constructed using the NEBNext Ultra DNA Library Prep Kit for Illumina (NEB, USA) and Single Molecule, Real-Time (SMRT) bell TM Template kit (version 2.0). Sequencing was performed at Beijing Novogene Bioinformatics Technology Co., Ltd. The low-quality reads were filtered with SMRT Link v8.0. The filtered reads were assembled using the Falcon software to generate contigs. The genome of the strain DM1 was accessible at GenBank (accession number JBGDMF000000000.1, GCA_050948135.1).

### Plasmid construction

The *hog1* gene was disrupted through homologous recombination, utilizing the hygromycin B phosphotransferase (*hph*) gene as a selectable marker. To construct the knockout vector, two 2 kb homologous arms corresponding to the 5′ and 3′ flanking regions of the *hog1* gene were amplified by polymerase chain reaction (PCR) using *A. sydowii* DM1 genomic DNA as template. The hygromycin-B resistance fragment was amplified using ko-pjet-hyg (6,720 bp) as the template. These PCR fragments were subsequently assembled into the pUC19 vector using the One Step Fusion Cloning Mix (TOROIVD, Shanghai), generating the final knockout plasmid pUC19-ashog1-ko (6,039 bp). The integrity of the recombinant plasmid was verified through PCR amplification and Sanger sequencing. The primer sequences used in this study are provided in supplementary material (Table S9). For genetic complementation, G418 (Geneticin) was employed as a selection marker at a concentration of 600 mg/mL. The mutant generated by the homologous recombination was transformed with a plasmid containing the G418-resistance gene and full-length *hog1* gene including the regulatory regions (promoter and terminator). The successful integration of the complementation construct was confirmed by PCR analysis. The complementation plasmid was constructed following the same methodology as the knockout plasmid.

### Preparation of protoplasts

Fungal transformations were performed using a polyethylene glycol (PEG)-mediated method, as previously described by Turgeon et al. (64). (the composition of all solutions is shown in Table S8). Briefly, fungal spore suspensions (100 µL, 10^6^ spores /μL) were inoculated into 250 mL of potato dextrose broth (PDB) and incubated in a shaker (100 rpm) at 28°C for 3 days. Subsequently, mycelia were harvested by filtration through three layers of sterile gauze and transferred to a 100 mL sterilized flask. A total of 20 mL of Solution 2 containing 0.02 g Yatalase (Takara), 0.03 g Vinotaste (Novozymes), and 0.02 g Snailase (BBI) were added. All enzyme solutions were filtered through a 0.22 µm filter to sterilize prior to use. The mixture was incubated at 30°C shaking at 100 × *g* for 2 h. The enzymatically digested suspension was then filtered through sterile gauze, and an equal volume of Solution 5 was gently added. The suspension was centrifuged at 5,000 × *g* for 10 min at 4°C. The white intermediate layer, containing the protoplasts, was carefully collected, washed with Solution 6 to remove impurities, and resuspended in 1 mL of Solution 7 for further use. The solutions used in this study were shown in the supplementary material (Table S8).

### Protoplast transformation of *A. sydowii*

Protoplasts (160 µL, 10⁶–10⁷ cells/mL) were mixed with 40 µL of Solution 8 (Table S8), followed by the addition of 20 µL of recombinant DNA fragments (concentration ≥100 ng/µL). The mixture was gently resuspended by pipetting and incubated on ice for 1 h. Subsequently, 1 mL of Solution 8 was added, and the suspension was gently mixed by pipetting, followed by incubation at room temperature for 30 min. The entire suspension was then transferred to a 15 mL centrifuge tube, and 4 mL of Solution 7 was added. The tube was inverted several times to ensure thorough mixing. Next, 10 mL of PDB containing selective pressure was added, and the mixture was gently inverted to homogenize. Aliquots (5 mL) were plated onto selection media PDA supplemented with 0.2 M NaCl and 100 µg/mL hygromycin B). The plates were rotated to ensure even distribution of the suspension and incubated at 28°C for 3–4 days. Finally, the resulting transformants were selected for verification by PCR and Southern blotting.

### Preparation of spore suspension and HHP treatment of fungal spores

Purified fungal hyphae were inoculated onto PDA plates and incubated at 28°C for 7 days. Colonies of uniform size and morphology were selected and transferred to a 50 mL centrifuge tube. Sterile purified water was added, and the mixture was vortexed thoroughly to remove spores from the hyphae. The resulting suspension was filtered through sterile gauze to remove residual hyphae, yielding a purified spore suspension. The spore concentration was quantified using a hemocytometer under a microscope, and the suspension was adjusted to a uniform density of 1 × 10⁶ spores/mL by adding sterile purified water. A 2 mL aliquot of the spore suspension was transferred into a sterile syringe and introduced into a high-pressure chamber filled with pure water. After comparing with previous research experiments, the pressure setting values of 20–80 MPa for this study have been determined (18, 19). The chamber was pressurized to various hydrostatic pressures (0.1, 20, 40, 60, and 80 MPa) and maintained at 25°C for 24 h. After treatment, the spore suspension was collected for subsequent experiments.

### Extraction of secondary metabolites, analysis of HPLC, and antibacterial verification

An appropriate amount of spore suspension was added to rice solid medium and cultured at 28°C for 14 days. After incubation, the rice was soaked in ethyl acetate to extract secondary metabolites. The ethyl acetate extract was evaporated at 40°C to yield the crude extract, which was subsequently dissolved in methanol for analysis using high-performance liquid chromatography (HPLC) (Agilent). The HPLC analysis was performed using an Agilent Zorbax SB-C18 (4.6 × 250 mm, 5 µm) with gradient elution. The mobile phase consisted of water (solvent A) and methanol (solvent B) at a flow rate of 1 mL/min. The column temperature was maintained at 40°C, and detection was carried out at wavelength of 220 nm. All solvents used for HPLC analysis, including the mobile phase components, were purchased from Sinopharm Chemical Reagent Co., Ltd. (Shanghai, China).

The indicator bacteria were obtained from Shanghai Rainbowfish Company. Antibacterial activity testing was performed according to the method described by Xiao et al (65). Suspensions of three pathogenic bacteria—*Salmonella choleraesuis*, *Enterococcus faecalis*, and *Staphylococcus aureus*, were evenly spread onto Luria-Bertani (LB) agar plates. Methanol was used as a negative control. The crude extract was dissolved in methanol to a final concentration of 100  mg/mL. Filter paper discs impregnated with the crude extract were placed in the center of the plates, which were then incubated at 37°C for 12 h.

### Effect of H_2_O_2_ on the growth of fungal plate and fluorescence analysis of ROS

When preparing culture plates with different concentrations of H₂O₂, the medium should be allowed to cool slightly after sterilization to minimize the risk of rapid H₂O₂ decomposition. Once the medium has reached an appropriate temperature, H₂O₂ (3%, Macklin) can be added. Subsequently, 1 µL of the spore suspension was inoculated onto the center of each plate and incubated at 28°C for 3 days.

The spore suspension which was used to measure the ROS content was first treated in the corresponding pressure group for 10 h. For the determination of ROS production in spore suspension, samples were treated with 10 µM 2′,7′-dichlorodihydrofluorescein diacetate (DCFH-DA) (Beyotime, Shanghai) according to the manufacturer’s instructions. After 4 h of incubation in the dark at room temperature (25°C), the fluorescence intensity of the oxidized product, dichlorofluorescein (DCF), was measured using a fluorescent enzyme-linked immunosorbent assay reader (TECAN, Shanghai) with an excitation/emission wavelength of 490/525 nm.

The spore suspension was mixed with PDB at a ratio of 1:1, and the spores were incubated at 28°C for 24 h to allow germination, thereby obtaining mycelium. The hyphae were then treated with 10 µM DCFH-DA. After 4 h of incubation, images were captured under green fluorescence using a microscope (OLYMPUS, BX53).

### Measurement of metabolic activity and cell viability

The resazurin assay, as described in previous reports, was utilized to assess the metabolic activity of spores (44). Three different salt concentrations (0, 1, and 4 mol/L) of resazurin solution were prepared by dissolving in PBS (ThermoFisher). Fifty microliters of each salt-resazurin solution and 50 µL of spore suspension (1 × 10⁶ spores/mL) were added to a 96-well plate and mixed, resulting in a final volume of 100 µL and a final resazurin concentration of 25 µg/mL. The absorbance was measured every 24 h at room temperature (25°C) using an ELISA reader.

Cell viability was assessed using Bac-Titer Lumi Plus (Beyotime, Shanghai) according to the manufacturer’s protocol. The spore suspension was obtained from plate containing varying concentrations of NaCl in double-distilled water and incubated for 12 h prior to analysis. Fifty microliters of spore suspension and 50 µL of the detection reagent were added to each well of an opaque 96-well plate. Luminescence was measured using an ELISA reader, and the results were recorded after the signal had stabilized.

### Statistical analysis

All experiments and groups were conducted at least in biological triplicates, and then three technical replicates were measured each time. The differences between the variables were evaluated by one-way analysis of variance (ANOVA), followed by Tukey’s multiple comparison test. Results were considered significantly different at *P* < 0.05 (*P < 0.05, **P < 0.01, ***P < 0.001). Graphs and statistical calculations were performed using GraphPad Prism 8 (GraphPad software version 8.0.2, San Diego, CA). The KEGG pathway was drawn using Weishengxin (https://www.bioinformatics.com.cn/).

## Data Availability

All data used for this study have been included in the article or supplemental material.

## SUPPLEMENTAL MATERIAL

Supplemental material aem.00921-25-s0001.docx Fig. S1 to S8; Tables S1 to S9.

ASM does not own the copyrights to Supplemental Material that may be linked to, or accessed through, an article. The authors have granted ASM a non-exclusive, world-wide license to publish the Supplemental Material files. Please contact the corresponding author directly for reuse.
