# Supplementary material for: The HOG signal pathway contributes to survival strategies of the piezo-tolerant fungus Aspergillus sydowii DM1 in hadal sediments
Source: Appl Environ Microbiol. 2025 Aug 12;91(9):e00921-25. doi: 10.1128/aem.00921-25 (PMC12442359; doi:10.1128/aem.00921-25)
Supplement: Supplemental material — Fig. S1 to S8; Tables S1 to S9. [file aem.00921-25-s0001.docx]

**The HOG signal pathway contributes to** **survival strategies of the piezo-tolerant fungus, *Aspergillus sydowii* DM1, in hadal sediments**

Guangzhao Hu^a^, Maosheng Zhong^a^, Changhao Zhang^a^, Hongfu Lai^a^, Eva Breyer^a^, Jiasong Fang^1^, Xi Yu^1#^

Guangzhao Hu and Maosheng Zhong contributed equally to this work

^a^ Shanghai Engineering Research Center of Hadal Science and Technology, College of Oceanography and Ecological Science, Shanghai Ocean University, Shanghai, 201306, China

Guangzhao Hu ORCID: https://orcid.org/0000-0002-5019-9391

*Corresponding author: XY (x[yu@shou.edu.cn](mailto:yu@shou.edu.cn)) ORCID: http://orcid.org/0000-0003-4243-6016

Lead contact: Xi Yu

**Running title**: HOG pathway helps fungi survival in hadal sediments

**Supplementary Figure S1**


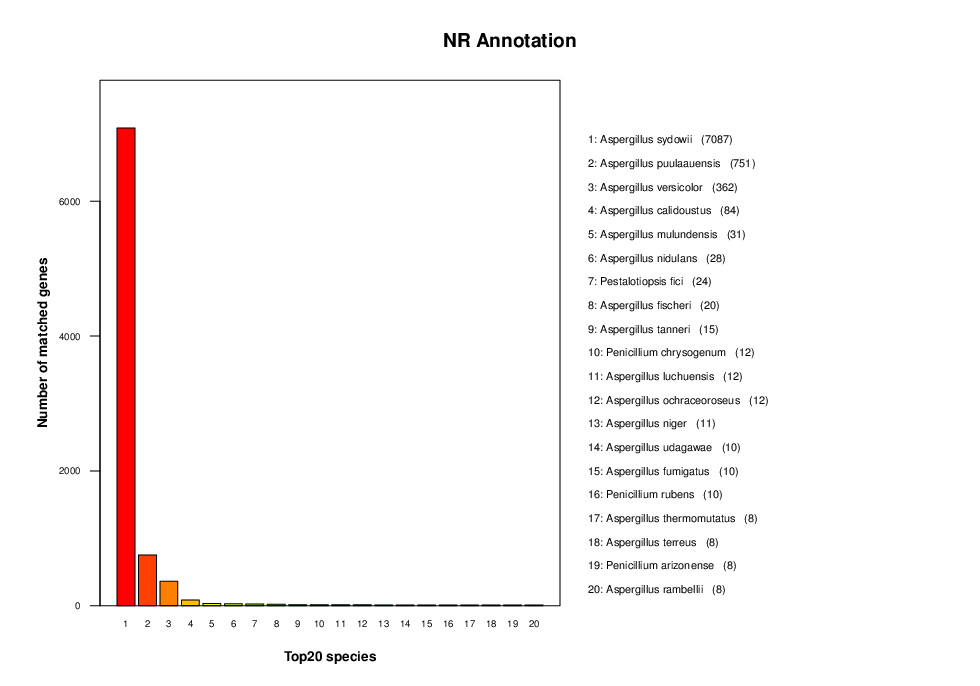


**Supplementary Figure S1.** Statistics of annotation results of NR database.


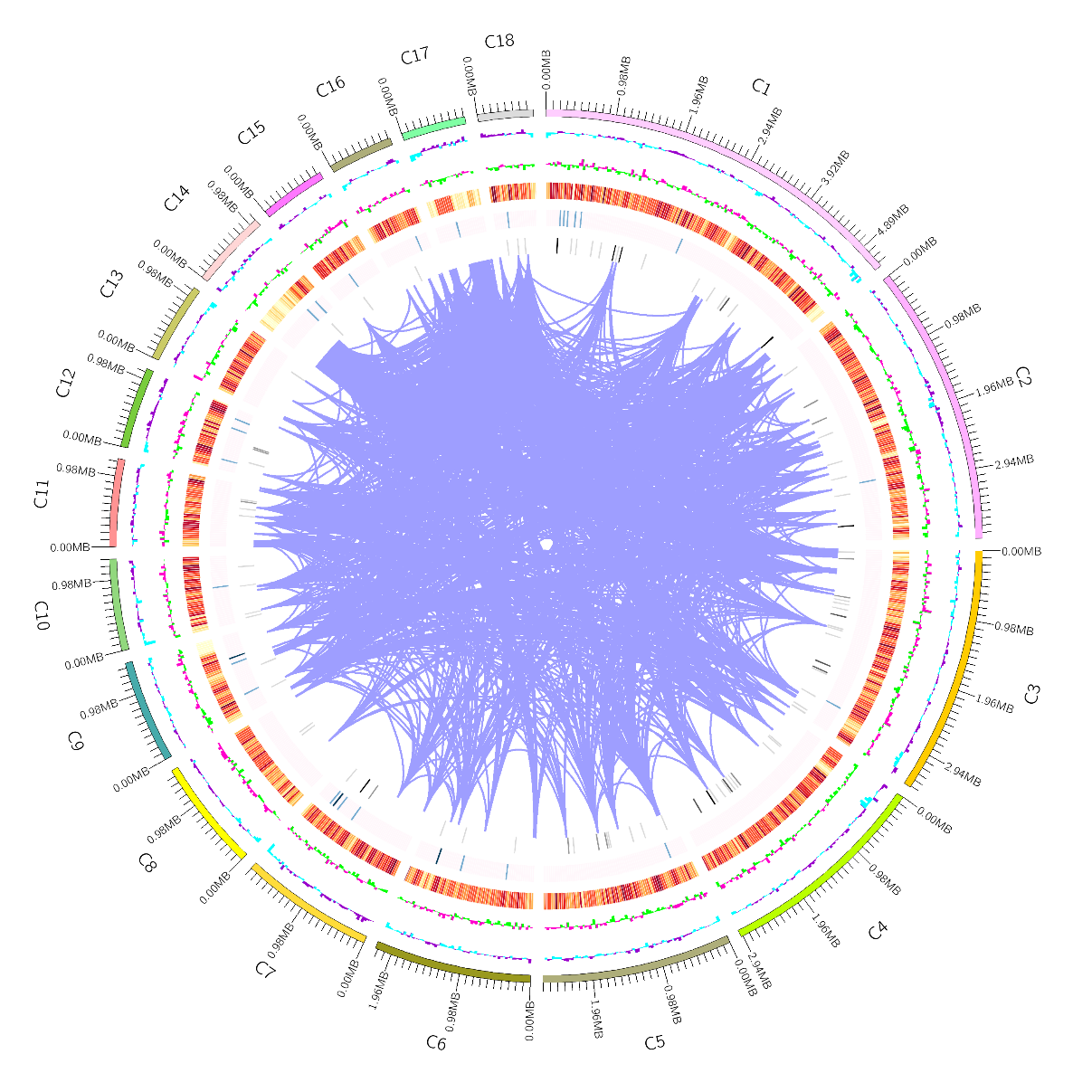
**Supplementary Figure S2**

**Supplementary Figure S2.** From outer to inner rings, the data shown are GC content, GC-skew, gene density (including coding genes, rRNA, snRNA, and tRNA), and gene duplication. The outermost ring represents the genomic coordinates. The GC content of the genome is calculated using a sliding window of size (chromosome length/1000) bp with a step size of (chromosome length/1,000) bp. The inward cyan regions indicate areas where the GC content is lower than the average GC content of the whole genome, while the outward dark purple regions represent areas where the GC content is higher than the average. The taller the peaks, the greater the difference from the average GC content. The GC skew values are also calculated using the same window and step size, following the formula (G-C)/(G+C). The inward light green areas show regions where the G content is lower than the C content, while the outward pink regions indicate the opposite. Gene density is represented by the proportion of genes in each window, including coding genes, rRNA, snRNA, and tRNA. Finally, the innermost ring displays the distribution of gene duplications across the genome.

**Supplementary Figure S3**


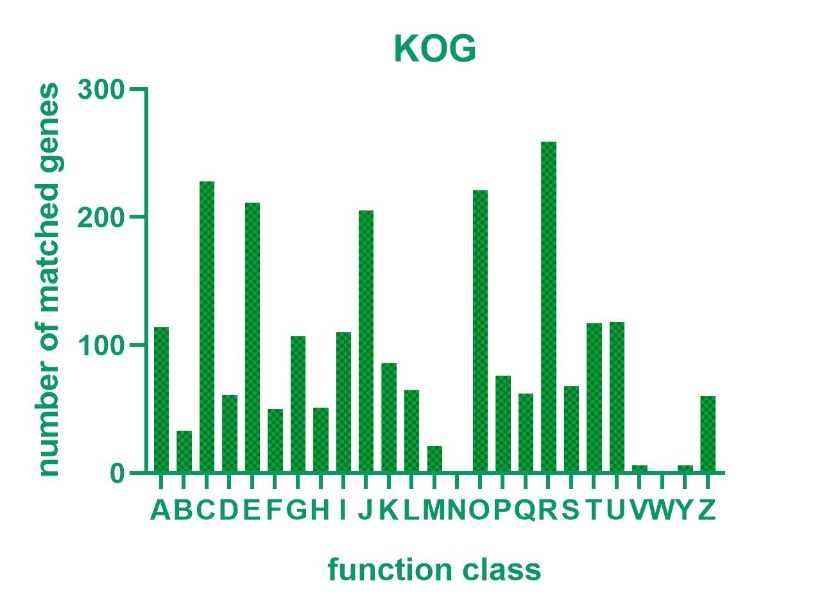


**Supplementary Figure S3.** KOG functional classification of DM1.

A RNA processing and modification 114

B Chromatin structure and dynamics 33

C Energy production and conversion 228

D Cell cycle control, cell division, chromosome partitioning 61

E Amino acid transport and metabolism 211

F Nucleotide transport and metabolism 50

G Carbohydrate transport and metabolism 107

H Coenzyme transport and metabolism 51

I Lipid transport and metabolism 110

J Translation, ribosomal structure and biogenesis 205

K Transcription 86

L Replication, recombination and repair 65

M Cell wall/membrane/envelope biogenesis 21

N Cell motility 1

O Posttranslational modification, protein turnover, chaperones 221

P Inorganic ion transport and metabolism 76

Q Secondary metabolites biosynthesis, transport and catabolism 62

R General function prediction only 259

S Function unknown 68

T Signal transduction mechanisms 117

U Intracellular trafficking, secretion, and vesicular transport 118

V Defense mechanisms 6

W Extracellular structures 1

Y Nuclear structure 6

Z Cytoskeleton 60

**Supplementary Figure S4**


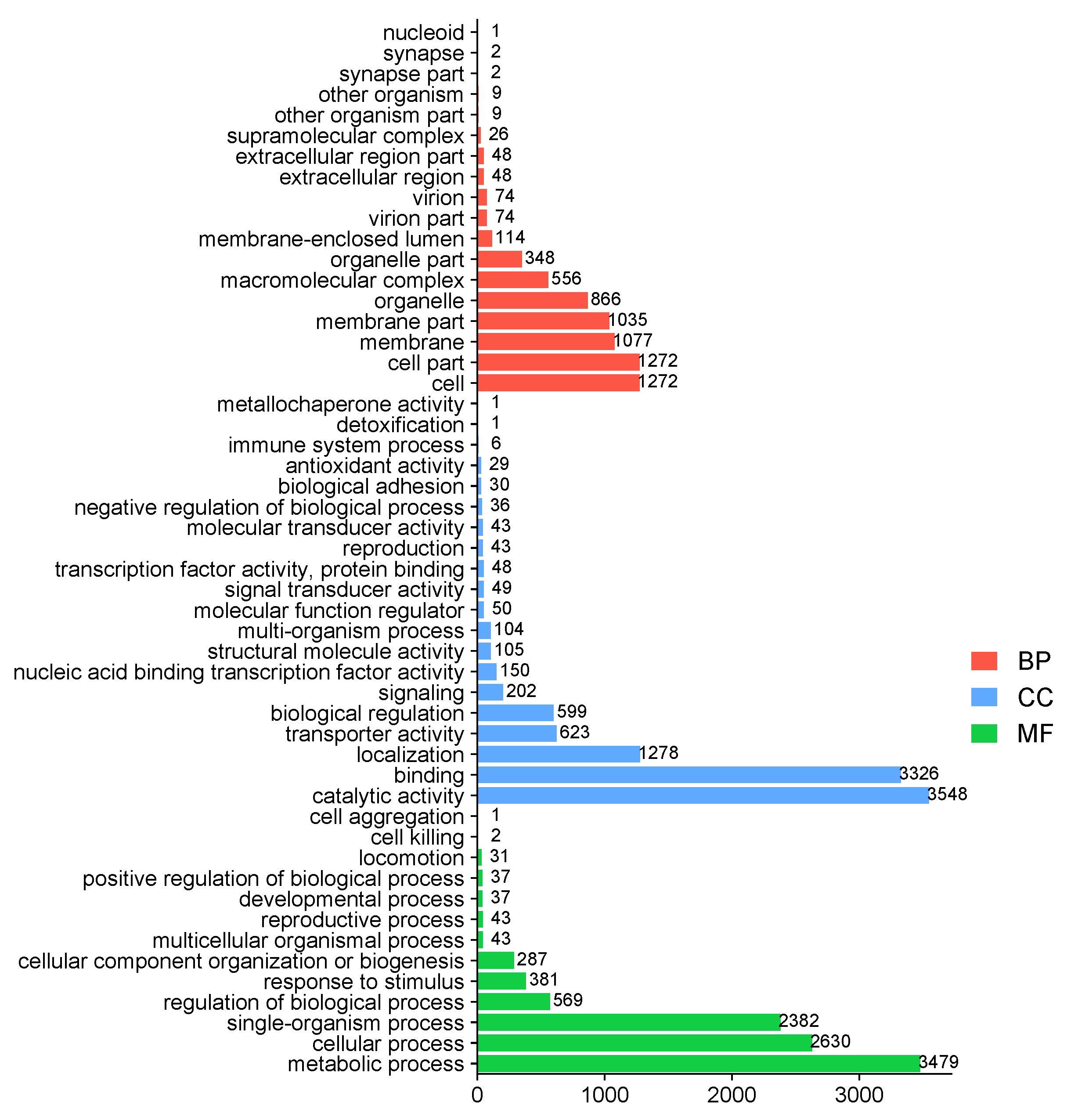

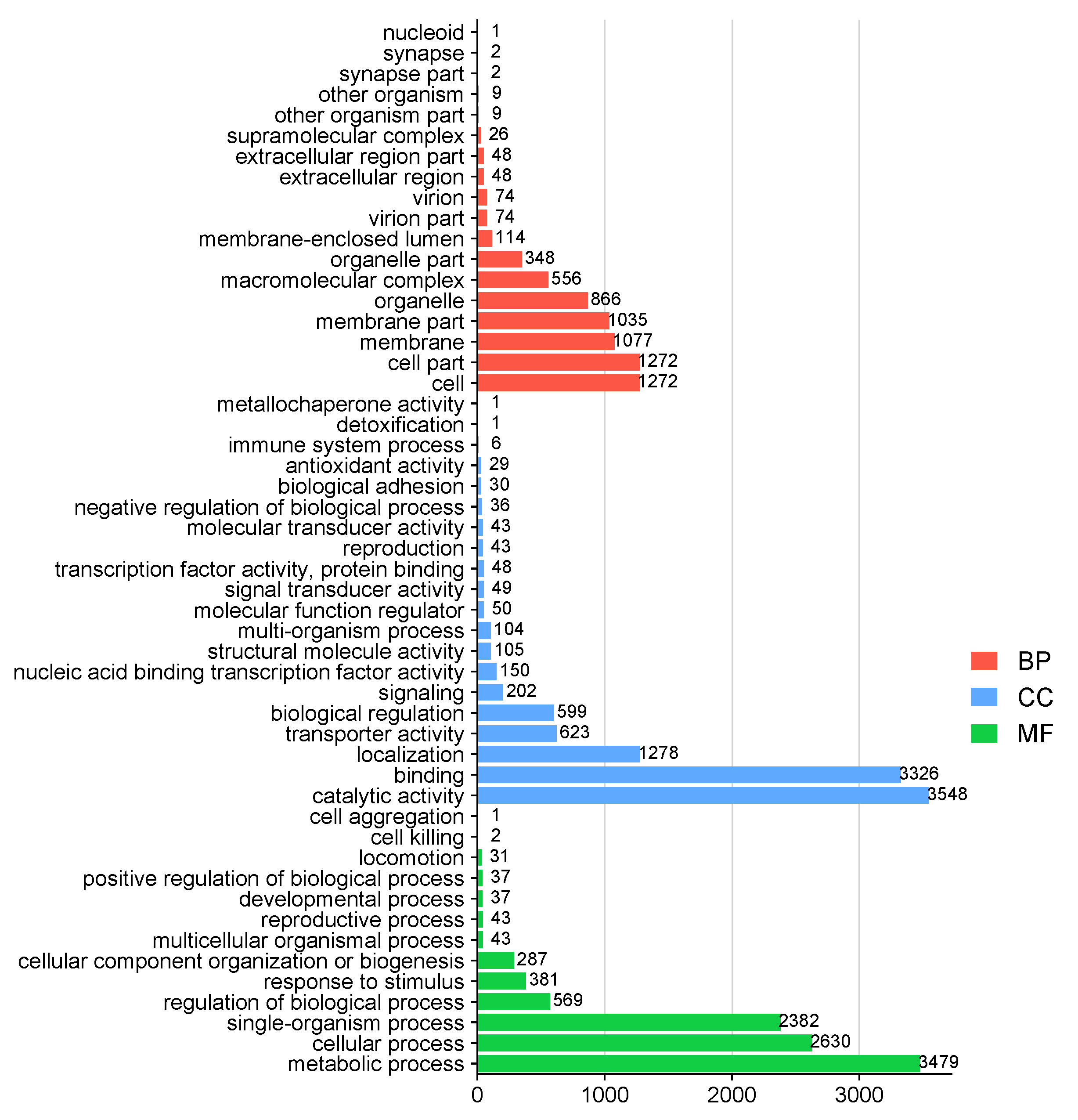


: Biological Process

: Cellular Component

: Molecular Function

**Supplementary Figure S4.** GO functional classification of DM1.


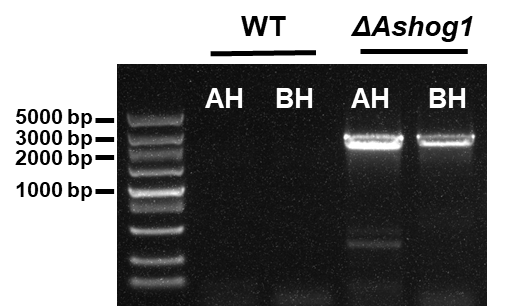

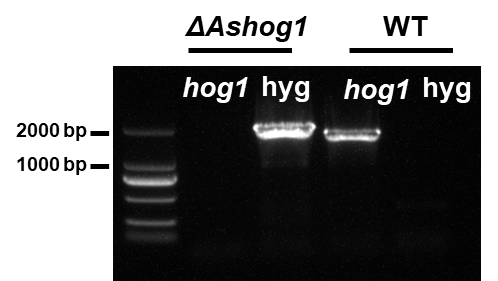
**Supplement Figure S5**

**Supplement Figure S5.** Mutant strain’s PCR validation results. The results showed that the *hog1* gene fragment was missing in the knockout strain.

**Supplementary Figure** **S6**


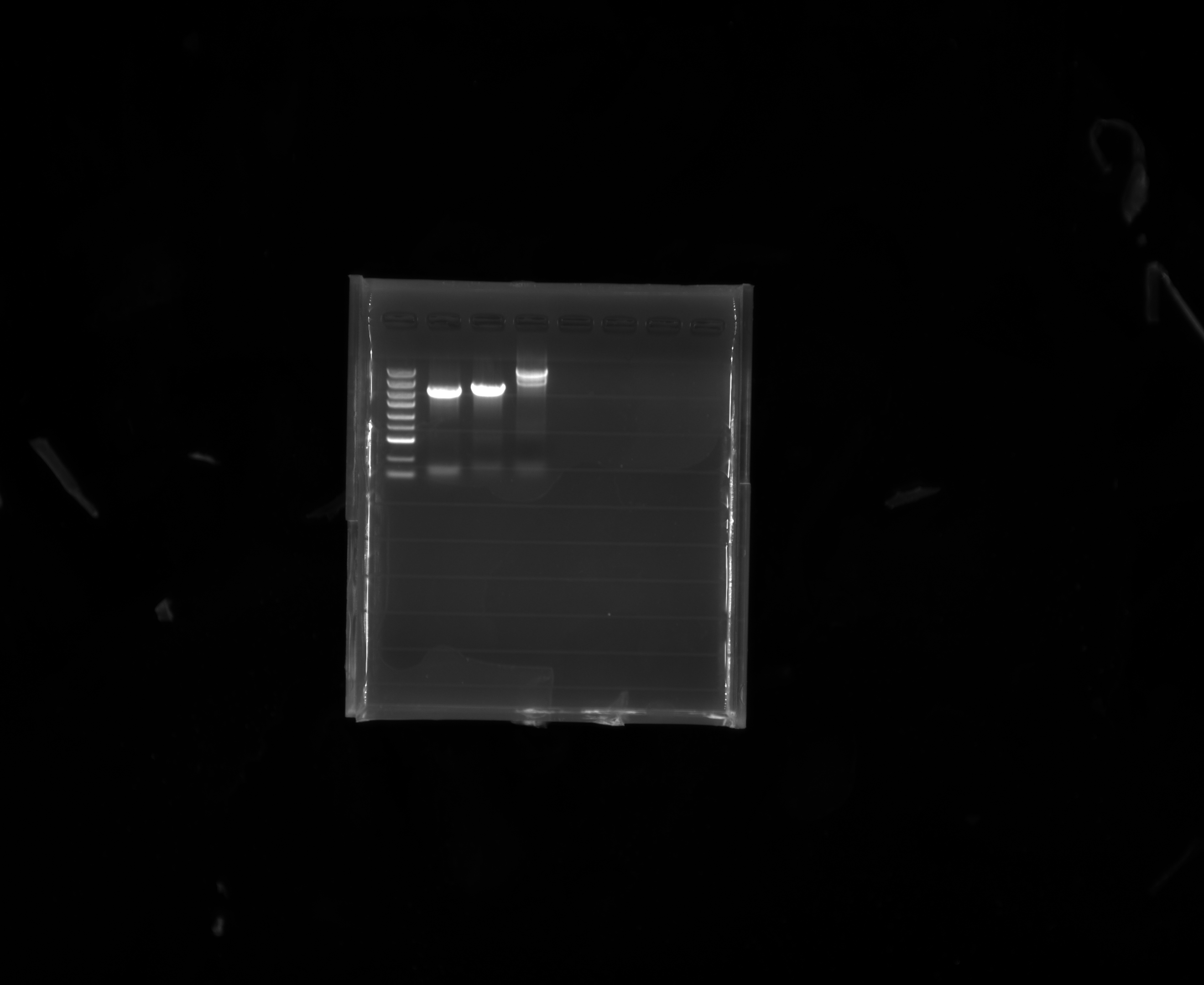


*hog1*

*G418*

*hog1+G418*

*2000bp*

*5000bp*

**Supplementary Figure S6** The PCR verification of the complement strain showed clear amplification of the *hog1* fragment, confirming its integration into the strain's genome.

**Supplementary Figure** **S7**

A


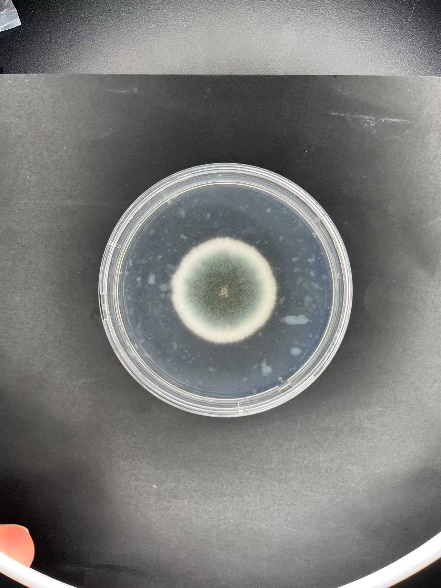

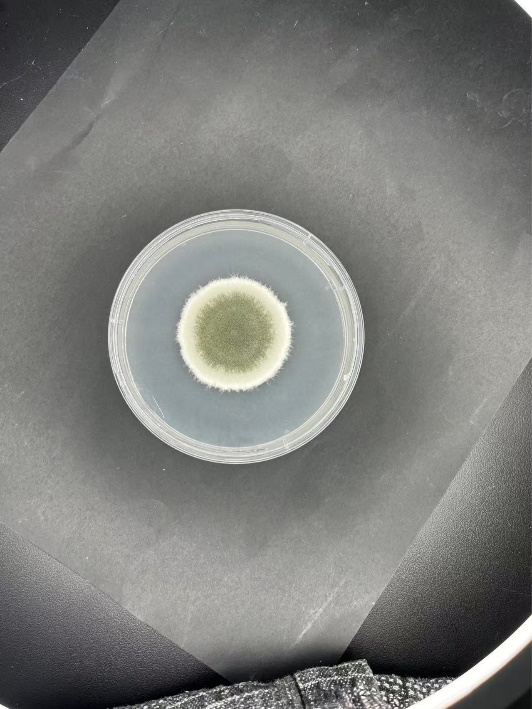

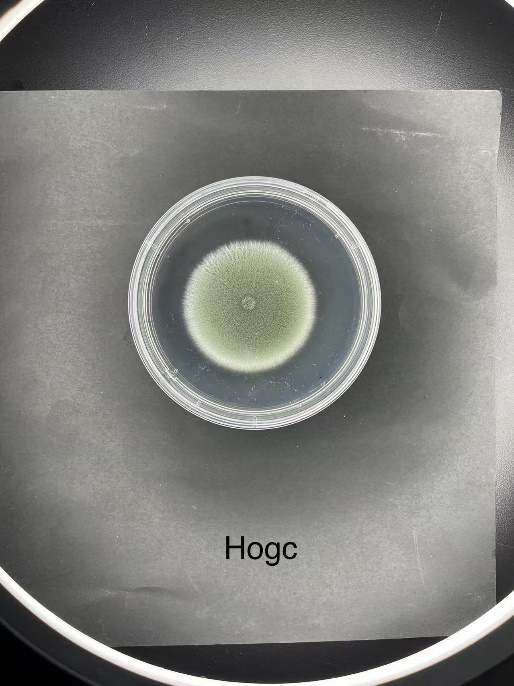


WT

Δ*Ashog1*

Δ*Ashog1-C*


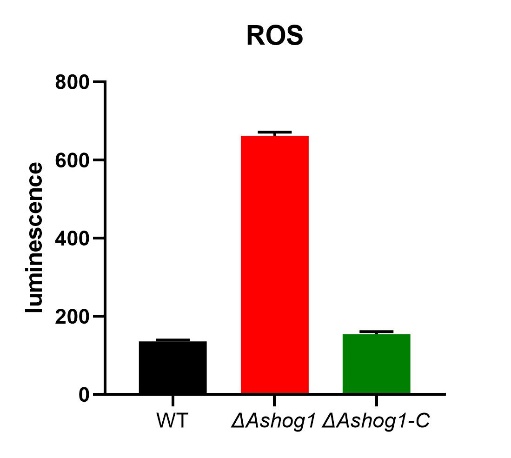


B

**Supplementary Figure** **S7** The phenotypes of the complement strain and the recovery of ROS levels are basically the same as those of the WT. **A** WT, knockout strains and complement strains grown in PDA for 7days. **B** ROS values of spore suspensions of WT, knockout strains and complem ent strains grown on 0.5 M NaCl PDA for 7 days.

**Supplementary Figure S8**


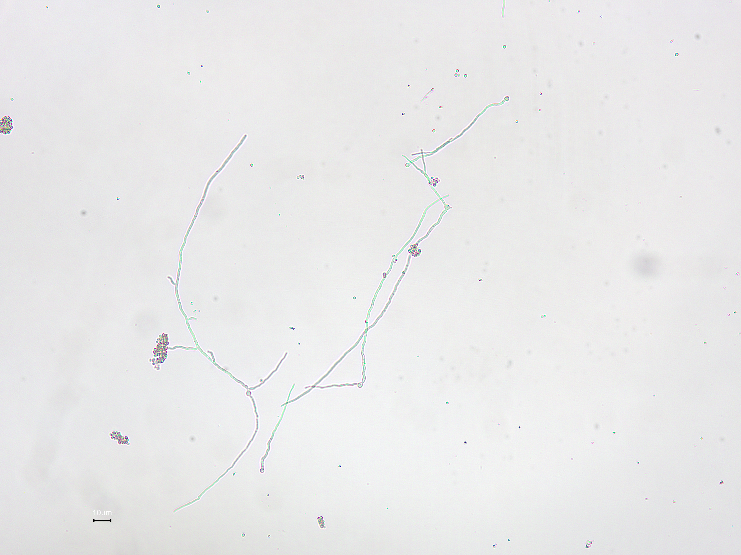


10mm

WT


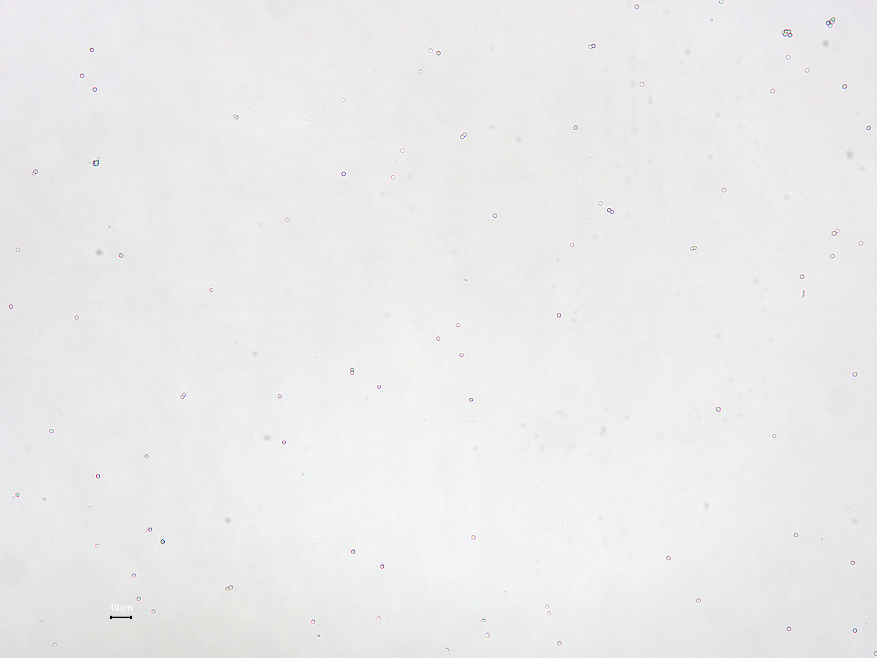


10mm

Δ*Ashog1*

**Supplementary Figure S8.** Microscopic images of spore germination after 24-hour treatment with 80 MPa were obtained. Notably, spores of the WT strain exhibited extensive germination under 80 MPa for 24 hours, whereas no germination was observed in the Δ*Ashog1* mutant spores.

| **Table S1** Genome data of DM1 | |
| --- | --- |
| **Sample. ID** | **DM1** |
| Mean.Concordance | 0.88 |
| Number.of.Bases.bp. | 18,265,533,643 |
| Number.of.Reads | 3,054,138 |
| Mean.Read.Length.bp. | 5,980 |
| N50.Read. Length.bp. | 8,982 |
| Contigs | 21 |
| Max_Length.bp. | 5,244,598 |
| N50_Length.bp | 2,651,975 |
| Total_length.bp. | 35,335,258 |
| GC% | 50.57 |
| Genome.size.bp. | 35,335,258 |
| Gene.number. | 12,241 |

| **Table S2** Results of BUSCO evaluation of coding genes | | |
| --- | --- | --- |
| **Sample. ID** | **DM1** |  |
| C | 99.30% | Complete BUSCOs |
| S | 98.30% | Complete and single-copy BUSCOs |
| D | 1.00% | Complete Duplicated BUSCOs |
| F | 0.00% | Fragmented BUSCOs |
| M | 0.70% | Missing BUSCOs |
| n | 290 | Total BUSCO groups searched |

| **Table S3.** Statistics of annotation results of repeat sequence | | | | |
| --- | --- | --- | --- | --- |
| **Type** | **Number** | **Total Length bp** | **In genome** | **Average length bp** |
| LTR | 906 | 126,935 | 0.3592 | 143 |
| DNA | 520 | 123,994 | 0.3509 | 241 |
| LINE | 288 | 90,109 | 0.255 | 316 |
| SINE | 68 | 5,286 | 0.015 | 78 |
| RC | 26 | 1,792 | 0.0051 | 69 |
| Unknown | 7 | 506 | 0.0014 | 72 |
| Total | 1,815 | 344,053 | 0.9737 | 195 |

| **Table S4** KEGG pathway of fungus DM1 and two other strains of *A. sydowii* | | | |
| --- | --- | --- | --- |
| **Pathway** | **DM1** | **BOBA1** | **CBS593.65** |
| Metabolism | 2,974 | 3,322 | 3,060 |
| Global and overview maps | 1,597 | 1,795 | 1,648 |
| Carbohydrate metabolism | 303 | 341 | 312 |
| Energy metabolism | 135 | 126 | 137 |
| Lipid metabolism | 158 | 170 | 163 |
| Nucleotide metabolism | 71 | 77 | 71 |
| Amino acid metabolism | 280 | 318 | 282 |
| Metabolism of other amino acids | 55 | 66 | 56 |
| Glycan biosynthesis and metabolism | 85 | 90 | 89 |
| Metabolism of cofactors and vitamins | 125 | 149 | 133 |
| Metabolism of terpenoids and polyketides | 28 | 30 | 29 |
| Biosynthesis of other secondary metabolites | 63 | 68 | 65 |
| Xenobiotics biodegradation and metabolism | 74 | 80 | 75 |
| Genetic Information Processing | 849 | 847 | 915 |
| Transcription | 117 | 114 | 137 |
| Translation | 263 | 266 | 292 |
| Folding, sorting and degradation | 234 | 231 | 246 |
| Replication and repair | 148 | 149 | 152 |
| Chromosome | 70 | 70 | 71 |
| Information processing in viruses | 17 | 17 | 17 |
| Environmental Information Processing | 410 | 414 | 445 |
| Membrane transport | 9 | 10 | 9 |
| Signal transduction | 397 | 400 | 432 |
| Signaling molecules and interaction | 4 | 4 | 4 |
| Cellular Processes | 762 | 759 | 783 |
| Transport and catabolism | 361 | 366 | 374 |
| Cell growth and death | 287 | 278 | 292 |
| Cellular community - eukaryotes | 50 | 50 | 53 |
| Cellular community - prokaryotes | 12 | 13 | 13 |
| Cell motility | 52 | 52 | 51 |
| Organismal Systems | 758 | 771 | 791 |
| Immune system | 160 | 164 | 173 |
| Endocrine system | 183 | 181 | 189 |
| Circulatory system | 34 | 33 | 33 |
| Digestive system | 51 | 55 | 52 |
| Excretory system | 37 | 37 | 38 |
| Nervous system | 127 | 134 | 130 |
| Sensory system | 8 | 8 | 8 |
| Development and regeneration | 29 | 29 | 31 |
| Aging | 44 | 42 | 45 |
| Environmental adaptation | 85 | 88 | 92 |
| Human Diseases | 1,805 | 1,853 | 1,873 |
| Cancer: overview | 203 | 212 | 213 |
| Cancer: specific types | 101 | 110 | 110 |
| Infectious disease: viral | 261 | 261 | 288 |
| Infectious disease: bacterial | 223 | 225 | 231 |
| Infectious disease: parasitic | 22 | 24 | 22 |
| Immune disease | 21 | 21 | 22 |
| Neurodegenerative disease | 720 | 734 | 726 |
| Substance dependence | 35 | 35 | 35 |
| Cardiovascular disease | 99 | 105 | 102 |
| Endocrine and metabolic disease | 82 | 85 | 85 |
| Drug resistance: antimicrobial | 1 | 2 | 1 |
| Drug resistance: antineoplastic | 37 | 39 | 38 |

| **Table S5** Unique genes of DM1 after cluster analysis with other two fungi | | | | | | |
| --- | --- | --- | --- | --- | --- | --- |
| **Gene name** | **E-value** | **COG_category** | **Description** | **KEGG_ko** | **BRITE** | **PFAMs** |
| Contig1.g1,513 | 0 | P | HCO_3_^-^ transporter family | ko:K02355 | ko00000,ko03012,ko03029 | HCO_3_^-^ cotransp |
| Contig3.g3,157 | 2.47E-20 | M | Ankyrin repeat | - | - | Ank_2 |
| Contig3.g3,279 | 4.99E-121 | - | - | - | - | Pkinase,Pkinase_Tyr |
| Contig3.g4,213 | 1.05E-148 | G | Lysin motif | - | - | LysM |
| Contig3.g4,218 | 3.35E-117 | - | - | - | - | Pkinase,Pkinase_Tyr |
| Contig4.g4,430 | 4.63E-103 | S | LysM domain | - | - | LysM |
| Contig4.g4,442 | 0 | S | Phosphotransferase enzyme family | - | - | APH |
| Contig4.g4,443 | 3.64E-86 | - | - | - | - | - |
| Contig4.g4,448 | 0 | S | Protein of unknown function (DUF3435) | ko:K15116 | ko00000,ko02000 | DUF3435 |
| Contig6.g6,274 | 7.94E-30 | - | - | - | - | - |
| Contig6.g6,290 | 2.23E-68 | - | - | - | - | - |
| Contig9.g8,509 | 1.04E-170 | - | - | - | - | Pkinase,Pkinase_Tyr |
| Contig9.g8,513 | 5.45E-24 | M | Ankyrin repeat | - | - | Ank_2 |
| Contig11.g9,176 | 2.97E-170 | - | - | - | - | Pkinase,Pkinase_Tyr |
| Contig14.g10,422 | 7.94E-30 | - | - | - | - | - |
| Contig14.g10,438 | 6.66E-15 | M | Ankyrin repeat | ko:K15502,ko:K15503,ko:K21440 | ko00000,ko01009,ko03400,ko04131 | Ank_2,Ank_3,Ank_4,Ank_5 |
| Contig14.g10,488 | 5.81E-68 | - | - | - | - | - |
| Contig14.g10,529 | 5.65E-48 | - | - | - | - | DUF3435 |
| Contig14.g10,530 | 2.10E-91 | S | Protein of unknown function (DUF3589) | ko:K13682 | ko00000,ko01000,ko01003 | DUF3589 |
| Contig14.g10,547 | 3.60E-35 | - | - | - | - | - |
| Contig14.g10,599 | 2.08E-32 | - | - | - | - | - |
| Contig14.g10,609 | 5.34E-61 | - | - | - | - | Zn_clus |
| Contig14.g10,610 | 2.23E-116 | - | - | - | - | - |
| Contig14.g10,614 | 3.60E-35 | - | - | - | - | - |
| Contig17.g11,518 | 0 | P | HCO_3_^-^ transporter family | ko:K02355 | ko00000,ko03012,ko03029 | HCO_3_^-^ cotransp |
| Contig17.g11,523 | 1.95E-49 | P | HCO_3_^-^ transporter family | ko:K02355 | ko00000,ko03012,ko03029 | HCO_3_^-^ cotransp |
| Contig17.g11,527 | 0 | P | HCO_3_^-^ transporter family | ko:K02355 | ko00000,ko03012,ko03029 | HCO_3_^-^ cotransp |
| Contig17.g11,592 | 0 | P | HCO_3_^-^ transporter family | ko:K02355 | ko00000,ko03012,ko03029 | HCO_3_^-^ cotransp |
| Contig17.g11,597 | 1.61E-292 | P | HCO_3_^-^ transporter family | ko:K02355 | ko00000,ko03012,ko03029 | HCO_3_^-^ cotransp |
| Contig17.g11,601 | 1.65E-68 | P | HCO_3_^-^ transporter family | ko:K02355 | ko00000,ko03012,ko03029 | HCO_3_^-^ cotransp |
| Contig17.g11,602 | 2.02E-257 | P | HCO_3_^-^ transporter family | ko:K02355 | ko00000,ko03012,ko03029 | HCO_3_^-^ cotransp |
| Contig17.g11,628 | 2.5e-314 | S | Protein of unknown function (DUF3435) | ko:K15116 | ko00000,ko02000 | DUF3435 |
| Contig17.g11,643 | 7.74E-28 | - | - | - | - | - |
| Contig17.g11,656 | 1.57E-120 | - | - | - | - | - |
| Contig17.g11,667 | 3.60E-35 | - | - | - | - | - |
| Contig20.g12,174 | 0 | S | Phosphotransferase enzyme family | - | - | APH |
| Contig20.g12,175 | 6.04E-85 | - | - | - | - | - |
| Contig20.g12,235 | 7.94E-30 | - | - | - | - | - |

| **Table S6** Primers used in the work | | | |
| --- | --- | --- | --- |
| **Primer** | **Sequences（5’-3’）** | **length (bp)** | **Function** |
| PUC19F1’ | ACTGGCCGTCGTTTTAC | 2,629 | vector construction |
| PUC19R1’ | GGCGTAATCATGGTCAT |  |  |
| HupF-1 | TAAAACGACGGCCAGTGATCCGACGAACAGGCAGAA | 704 | *hog1* upstream |
|  |  |  |  |
| HupR-1 | AGCATTACACTTGGATCCCGGCAGGTAGCAGTCCGAACA |  |  |
|  |  |  |  |
| HdownF-1 | TAAACTCCCCCCACTGCAGAACGCAGTAGGGACCTTGGAT | 849 | *hog1* downstream |
|  |  |  |  |
| HdownR-1 | AGCTATGACCATGATTACGCCAAGCAGGACAGAAGTGGATGAG |  |  |
|  |  |  |  |
| hyg-f | CGGGATCCAAGTGTAATGCTAGTGGA | 1,851 | *hph* resistant fragment |
| hyg-r | TTCTGCAGTGGGGGGAGTTTAGGGAAA |  |  |
| hog1F1 | CTATTGGAACCCTTGTTGATTCA | 1,654 | Objective fragment *hog1* |
| hog1R | ATGGCGGAATTCGTACGTGCT |  |  |
| probe-hygF | ATTCGGACCGCAAGGAAT | 752 | SB-validated resistant fragment insertion |
| probe-hygR | TGTCGCCCTTATTCGACTCA |  |  |
| probe-upF | ATGTCATTGAGTGCTTGACAA | 558 | S-Blotting Validation Upstream Fragment |
| probe-upR | TGACCGAGCGATAGCGG |  |  |

| **Table S7** Southern blotting validation fragment name and length | | |
| --- | --- | --- |
| **Strian name** | **Enzymolysis length** | **Probe length** |
| WT | 1,177 bp | 558 bp |
| Δ*Ashog1* | 570 bp |  |

| **Table S8** The culture medium/ reagents used in this study | | |
| --- | --- | --- |
| **Media** | **Composition** | **Remarks** |
| Potato dextrose agar (PDA) | potato 200 g/L, glucose 20 g/L, agar15 g/L | For plate culture of fungi |
| Potato dextrose broth (PDB) | potato 200 g/L, glucose 20 g/L | For liquid culture of fungi |
| Luria-Bertani (LB) broth | yeast extract 5 g/L, NaCl 10 g/L, peptone 10 g/L | For liquid culture of bacteria |
| Luria-Bertani (LB) agar | yeast extract 5 g/L, NaCl 10 g/L, peptone 10 g/L, agar 15 g/L | For plate culture of bacteria |
| Solution 2 | MgSO_4_·7H_2_O 298g/L, 0.2 M NaHPO_4_ 36mL/L, 0.2 M NaH_2_PO_4_ 1.4mL/L | For the transformation step in knockout experiments |
| Solution 5 | D-Sorbitol (0.6M) 109g/L, Tris-Cl 1 M 100ml/L | For the transformation step in knockout experiments |
| Solution 6 | D-Sorbitol (0.6M) 182.2g/L, Tris-Cl 1 M 10ml/L | For the transformation step in knockout experiments |
| Solution 7 | D-Sorbitol (0.6M) 182.2g/L, Tris-Cl 1 M 10ml/L, 1 M CaCl_2_ 10mL/L | For the transformation step in knockout experiments |
| Solution 8 | PEG 6000, Tris-Cl 1 M 5ml/L, 1 M CaCl_2_ 5mL/L | For the transformation step in knockout experiments |
| rice solid medium | rice400g, water 600g, NaCl 28 g/L | For metabolite extraction culture |

| **Table S9** PCR fragment name and length | | |
| --- | --- | --- |
| **Fragment name** | **Name abbreviation** | **Fragment length** |
| *hyg* | *hyg* | 1,854 bp |
| *hog1* | *hog1* | 1,654 bp |
| Up+*hyg* | AH | 2,555 bp |
| Down+*hyg* | BH | 2,700 bp |
| Up+*hog1*+Down | AB1 | 3,204 bp |
| Up+*hyg1*+Down | AB2 | 3,404 bp |
